# Performance of the ATLAS muon trigger in pp collisions at $\sqrt{s}=8$ TeV

**DOI:** 10.1140/epjc/s10052-015-3325-9

**Published:** 2015-03-13

**Authors:** ATLAS Collaboration, G. Aad, B. Abbott, J. Abdallah, S. Abdel Khalek, O. Abdinov, R. Aben, B. Abi, M. Abolins, O. S. AbouZeid, H. Abramowicz, H. Abreu, R. Abreu, Y. Abulaiti, B. S. Acharya, L. Adamczyk, D. L. Adams, J. Adelman, S. Adomeit, T. Adye, T. Agatonovic-Jovin, J. A. Aguilar-Saavedra, M. Agustoni, S. P. Ahlen, F. Ahmadov, G. Aielli, H. Akerstedt, T. P. A. Åkesson, G. Akimoto, A. V. Akimov, G. L. Alberghi, J. Albert, S. Albrand, M. J. Alconada Verzini, M. Aleksa, I. N. Aleksandrov, C. Alexa, G. Alexander, G. Alexandre, T. Alexopoulos, M. Alhroob, G. Alimonti, L. Alio, J. Alison, B. M. M. Allbrooke, L. J. Allison, P. P. Allport, J. Almond, A. Aloisio, A. Alonso, F. Alonso, C. Alpigiani, A. Altheimer, B. Alvarez Gonzalez, M. G. Alviggi, K. Amako, Y. Amaral Coutinho, C. Amelung, D. Amidei, S. P. Amor Dos Santos, A. Amorim, S. Amoroso, N. Amram, G. Amundsen, C. Anastopoulos, L. S. Ancu, N. Andari, T. Andeen, C. F. Anders, G. Anders, K. J. Anderson, A. Andreazza, V. Andrei, X. S. Anduaga, S. Angelidakis, I. Angelozzi, P. Anger, A. Angerami, F. Anghinolfi, A. V. Anisenkov, N. Anjos, A. Annovi, A. Antonaki, M. Antonelli, A. Antonov, J. Antos, F. Anulli, M. Aoki, L. Aperio Bella, R. Apolle, G. Arabidze, I. Aracena, Y. Arai, J. P. Araque, A. T. H. Arce, J-F. Arguin, S. Argyropoulos, M. Arik, A. J. Armbruster, O. Arnaez, V. Arnal, H. Arnold, M. Arratia, O. Arslan, A. Artamonov, G. Artoni, S. Asai, N. Asbah, A. Ashkenazi, B. Åsman, L. Asquith, K. Assamagan, R. Astalos, M. Atkinson, N. B. Atlay, B. Auerbach, K. Augsten, M. Aurousseau, G. Avolio, G. Azuelos, Y. Azuma, M. A. Baak, A. E. Baas, C. Bacci, H. Bachacou, K. Bachas, M. Backes, M. Backhaus, J. Backus Mayes, E. Badescu, P. Bagiacchi, P. Bagnaia, Y. Bai, T. Bain, J. T. Baines, O. K. Baker, P. Balek, F. Balli, E. Banas, Sw. Banerjee, A. A. E. Bannoura, V. Bansal, H. S. Bansil, L. Barak, S. P. Baranov, E. L. Barberio, D. Barberis, M. Barbero, T. Barillari, M. Barisonzi, T. Barklow, N. Barlow, B. M. Barnett, R. M. Barnett, Z. Barnovska, A. Baroncelli, G. Barone, A. J. Barr, F. Barreiro, J. Barreiro Guimarães da Costa, R. Bartoldus, A. E. Barton, P. Bartos, V. Bartsch, A. Bassalat, A. Basye, R. L. Bates, J. R. Batley, M. Battaglia, M. Battistin, F. Bauer, H. S. Bawa, M. D. Beattie, T. Beau, P. H. Beauchemin, R. Beccherle, P. Bechtle, H. P. Beck, K. Becker, S. Becker, M. Beckingham, C. Becot, A. J. Beddall, A. Beddall, S. Bedikian, V. A. Bednyakov, C. P. Bee, L. J. Beemster, T. A. Beermann, M. Begel, K. Behr, C. Belanger-Champagne, P. J. Bell, W. H. Bell, G. Bella, L. Bellagamba, A. Bellerive, M. Bellomo, K. Belotskiy, O. Beltramello, O. Benary, D. Benchekroun, K. Bendtz, N. Benekos, Y. Benhammou, E. Benhar Noccioli, J. A. Benitez Garcia, D. P. Benjamin, J. R. Bensinger, K. Benslama, S. Bentvelsen, D. Berge, E. Bergeaas Kuutmann, N. Berger, F. Berghaus, J. Beringer, C. Bernard, P. Bernat, C. Bernius, F. U. Bernlochner, T. Berry, P. Berta, C. Bertella, G. Bertoli, F. Bertolucci, C. Bertsche, D. Bertsche, M. I. Besana, G. J. Besjes, O. Bessidskaia, M. Bessner, N. Besson, C. Betancourt, S. Bethke, W. Bhimji, R. M. Bianchi, L. Bianchini, M. Bianco, O. Biebel, S. P. Bieniek, K. Bierwagen, J. Biesiada, M. Biglietti, J. Bilbao De Mendizabal, H. Bilokon, M. Bindi, S. Binet, A. Bingul, C. Bini, C. W. Black, J. E. Black, K. M. Black, D. Blackburn, R. E. Blair, J.-B. Blanchard, T. Blazek, I. Bloch, C. Blocker, W. Blum, U. Blumenschein, G. J. Bobbink, V. S. Bobrovnikov, S. S. Bocchetta, A. Bocci, C. Bock, C. R. Boddy, M. Boehler, T. T. Boek, J. A. Bogaerts, A. G. Bogdanchikov, A. Bogouch, C. Bohm, J. Bohm, V. Boisvert, T. Bold, V. Boldea, A. S. Boldyrev, M. Bomben, M. Bona, M. Boonekamp, A. Borisov, G. Borissov, M. Borri, S. Borroni, J. Bortfeldt, V. Bortolotto, K. Bos, D. Boscherini, M. Bosman, H. Boterenbrood, J. Boudreau, J. Bouffard, E. V. Bouhova-Thacker, D. Boumediene, C. Bourdarios, N. Bousson, S. Boutouil, A. Boveia, J. Boyd, I. R. Boyko, I. Bozic, J. Bracinik, A. Brandt, G. Brandt, O. Brandt, U. Bratzler, B. Brau, J. E. Brau, H. M. Braun, S. F. Brazzale, B. Brelier, K. Brendlinger, A. J. Brennan, R. Brenner, S. Bressler, K. Bristow, T. M. Bristow, D. Britton, F. M. Brochu, I. Brock, R. Brock, C. Bromberg, J. Bronner, G. Brooijmans, T. Brooks, W. K. Brooks, J. Brosamer, E. Brost, J. Brown, P. A. Bruckman de Renstrom, D. Bruncko, R. Bruneliere, S. Brunet, A. Bruni, G. Bruni, M. Bruschi, L. Bryngemark, T. Buanes, Q. Buat, F. Bucci, P. Buchholz, R. M. Buckingham, A. G. Buckley, S. I. Buda, I. A. Budagov, F. Buehrer, L. Bugge, M. K. Bugge, O. Bulekov, A. C. Bundock, H. Burckhart, S. Burdin, B. Burghgrave, S. Burke, I. Burmeister, E. Busato, D. Büscher, V. Büscher, P. Bussey, C. P. Buszello, B. Butler, J. M. Butler, A. I. Butt, C. M. Buttar, J. M. Butterworth, P. Butti, W. Buttinger, A. Buzatu, M. Byszewski, S. Cabrera Urbán, D. Caforio, O. Cakir, N. Calace, P. Calafiura, A. Calandri, G. Calderini, P. Calfayan, R. Calkins, L. P. Caloba, D. Calvet, S. Calvet, R. Camacho Toro, S. Camarda, D. Cameron, L. M. Caminada, R. Caminal Armadans, S. Campana, M. Campanelli, A. Campoverde, V. Canale, A. Canepa, M. Cano Bret, J. Cantero, R. Cantrill, T. Cao, M. D. M. Capeans Garrido, I. Caprini, M. Caprini, M. Capua, R. Caputo, R. Cardarelli, T. Carli, G. Carlino, L. Carminati, S. Caron, E. Carquin, G. D. Carrillo-Montoya, J. R. Carter, J. Carvalho, D. Casadei, M. P. Casado, M. Casolino, E. Castaneda-Miranda, A. Castelli, V. Castillo Gimenez, N. F. Castro, P. Catastini, A. Catinaccio, J. R. Catmore, A. Cattai, G. Cattani, J. Caudron, V. Cavaliere, D. Cavalli, M. Cavalli-Sforza, V. Cavasinni, F. Ceradini, B. C. Cerio, K. Cerny, A. S. Cerqueira, A. Cerri, L. Cerrito, F. Cerutti, M. Cerv, A. Cervelli, S. A. Cetin, A. Chafaq, D. Chakraborty, I. Chalupkova, P. Chang, B. Chapleau, J. D. Chapman, D. Charfeddine, D. G. Charlton, C. C. Chau, C. A. Chavez Barajas, S. Cheatham, A. Chegwidden, S. Chekanov, S. V. Chekulaev, G. A. Chelkov, M. A. Chelstowska, C. Chen, H. Chen, K. Chen, L. Chen, S. Chen, X. Chen, Y. Chen, Y. Chen, H. C. Cheng, Y. Cheng, A. Cheplakov, R. Cherkaoui El Moursli, V. Chernyatin, E. Cheu, L. Chevalier, V. Chiarella, G. Chiefari, J. T. Childers, A. Chilingarov, G. Chiodini, A. S. Chisholm, R. T. Chislett, A. Chitan, M. V. Chizhov, S. Chouridou, B. K. B. Chow, D. Chromek-Burckhart, M. L. Chu, J. Chudoba, J. J. Chwastowski, L. Chytka, G. Ciapetti, A. K. Ciftci, R. Ciftci, D. Cinca, V. Cindro, A. Ciocio, P. Cirkovic, Z. H. Citron, M. Citterio, M. Ciubancan, A. Clark, P. J. Clark, R. N. Clarke, W. Cleland, J. C. Clemens, C. Clement, Y. Coadou, M. Cobal, A. Coccaro, J. Cochran, L. Coffey, J. G. Cogan, J. Coggeshall, B. Cole, S. Cole, A. P. Colijn, J. Collot, T. Colombo, G. Colon, G. Compostella, P. Conde Muiño, E. Coniavitis, M. C. Conidi, S. H. Connell, I. A. Connelly, S. M. Consonni, V. Consorti, S. Constantinescu, C. Conta, G. Conti, F. Conventi, M. Cooke, B. D. Cooper, A. M. Cooper-Sarkar, N. J. Cooper-Smith, K. Copic, T. Cornelissen, M. Corradi, F. Corriveau, A. Corso-Radu, A. Cortes-Gonzalez, G. Cortiana, G. Costa, M. J. Costa, D. Costanzo, D. Côté, G. Cottin, G. Cowan, B. E. Cox, K. Cranmer, G. Cree, S. Crépé-Renaudin, F. Crescioli, W. A. Cribbs, M. Crispin Ortuzar, M. Cristinziani, V. Croft, G. Crosetti, C.-M. Cuciuc, T. Cuhadar Donszelmann, J. Cummings, M. Curatolo, C. Cuthbert, H. Czirr, P. Czodrowski, Z. Czyczula, S. D’Auria, M. D’Onofrio, M. J. Da Cunha Sargedas De Sousa, C. Da Via, W. Dabrowski, A. Dafinca, T. Dai, O. Dale, F. Dallaire, C. Dallapiccola, M. Dam, A. C. Daniells, M. Dano Hoffmann, V. Dao, G. Darbo, S. Darmora, J. A. Dassoulas, A. Dattagupta, W. Davey, C. David, T. Davidek, E. Davies, M. Davies, O. Davignon, A. R. Davison, P. Davison, Y. Davygora, E. Dawe, I. Dawson, R. K. Daya-Ishmukhametova, K. De, R. de Asmundis, S. De Castro, S. De Cecco, N. De Groot, P. de Jong, H. De la Torre, F. De Lorenzi, L. De Nooij, D. De Pedis, A. De Salvo, U. De Sanctis, A. De Santo, J. B. De Vivie De Regie, W. J. Dearnaley, R. Debbe, C. Debenedetti, B. Dechenaux, D. V. Dedovich, I. Deigaard, J. Del Peso, T. Del Prete, F. Deliot, C. M. Delitzsch, M. Deliyergiyev, A. Dell’Acqua, L. Dell’Asta, M. Dell’Orso, M. Della Pietra, D. della Volpe, M. Delmastro, P. A. Delsart, C. Deluca, S. Demers, M. Demichev, A. Demilly, S. P. Denisov, D. Derendarz, J. E. Derkaoui, F. Derue, P. Dervan, K. Desch, C. Deterre, P. O. Deviveiros, A. Dewhurst, S. Dhaliwal, A. Di Ciaccio, L. Di Ciaccio, A. Di Domenico, C. Di Donato, A. Di Girolamo, B. Di Girolamo, A. Di Mattia, B. Di Micco, R. Di Nardo, A. Di Simone, R. Di Sipio, D. Di Valentino, F. A. Dias, M. A. Diaz, E. B. Diehl, J. Dietrich, T. A. Dietzsch, S. Diglio, A. Dimitrievska, J. Dingfelder, C. Dionisi, P. Dita, S. Dita, F. Dittus, F. Djama, T. Djobava, M. A. B. do Vale, A. Do Valle Wemans, D. Dobos, C. Doglioni, T. Doherty, T. Dohmae, J. Dolejsi, Z. Dolezal, B. A. Dolgoshein, M. Donadelli, S. Donati, P. Dondero, J. Donini, J. Dopke, A. Doria, M. T. Dova, A. T. Doyle, M. Dris, J. Dubbert, S. Dube, E. Dubreuil, E. Duchovni, G. Duckeck, O. A. Ducu, D. Duda, A. Dudarev, F. Dudziak, L. Duflot, L. Duguid, M. Dührssen, M. Dunford, H. Duran Yildiz, M. Düren, A. Durglishvili, M. Dwuznik, M. Dyndal, J. Ebke, W. Edson, N. C. Edwards, W. Ehrenfeld, T. Eifert, G. Eigen, K. Einsweiler, T. Ekelof, M. El Kacimi, M. Ellert, S. Elles, F. Ellinghaus, N. Ellis, J. Elmsheuser, M. Elsing, D. Emeliyanov, Y. Enari, O. C. Endner, M. Endo, R. Engelmann, J. Erdmann, A. Ereditato, D. Eriksson, G. Ernis, J. Ernst, M. Ernst, J. Ernwein, D. Errede, S. Errede, E. Ertel, M. Escalier, H. Esch, C. Escobar, B. Esposito, A. I. Etienvre, E. Etzion, H. Evans, A. Ezhilov, L. Fabbri, G. Facini, R. M. Fakhrutdinov, S. Falciano, R. J. Falla, J. Faltova, Y. Fang, M. Fanti, A. Farbin, A. Farilla, T. Farooque, S. Farrell, S. M. Farrington, P. Farthouat, F. Fassi, P. Fassnacht, D. Fassouliotis, A. Favareto, L. Fayard, P. Federic, O. L. Fedin, W. Fedorko, M. Fehling-Kaschek, S. Feigl, L. Feligioni, C. Feng, E. J. Feng, H. Feng, A. B. Fenyuk, S. Fernandez Perez, S. Ferrag, J. Ferrando, A. Ferrari, P. Ferrari, R. Ferrari, D. E. Ferreira de Lima, A. Ferrer, D. Ferrere, C. Ferretti, A. Ferretto Parodi, M. Fiascaris, F. Fiedler, A. Filipčič, M. Filipuzzi, F. Filthaut, M. Fincke-Keeler, K. D. Finelli, M. C. N. Fiolhais, L. Fiorini, A. Firan, A. Fischer, J. Fischer, W. C. Fisher, E. A. Fitzgerald, M. Flechl, I. Fleck, P. Fleischmann, S. Fleischmann, G. T. Fletcher, G. Fletcher, T. Flick, A. Floderus, L. R. Flores Castillo, A. C. Florez Bustos, M. J. Flowerdew, A. Formica, A. Forti, D. Fortin, D. Fournier, H. Fox, S. Fracchia, P. Francavilla, M. Franchini, S. Franchino, D. Francis, L. Franconi, M. Franklin, S. Franz, M. Fraternali, S. T. French, C. Friedrich, F. Friedrich, D. Froidevaux, J. A. Frost, C. Fukunaga, E. Fullana Torregrosa, B. G. Fulsom, J. Fuster, C. Gabaldon, O. Gabizon, A. Gabrielli, A. Gabrielli, S. Gadatsch, S. Gadomski, G. Gagliardi, P. Gagnon, C. Galea, B. Galhardo, E. J. Gallas, V. Gallo, B. J. Gallop, P. Gallus, G. Galster, K. K. Gan, J. Gao, Y. S. Gao, F. M. Garay Walls, F. Garberson, C. García, J. E. García Navarro, M. Garcia-Sciveres, R. W. Gardner, N. Garelli, V. Garonne, C. Gatti, G. Gaudio, B. Gaur, L. Gauthier, P. Gauzzi, I. L. Gavrilenko, C. Gay, G. Gaycken, E. N. Gazis, P. Ge, Z. Gecse, C. N. P. Gee, D. A. A. Geerts, Ch. Geich-Gimbel, K. Gellerstedt, C. Gemme, A. Gemmell, M. H. Genest, S. Gentile, M. George, S. George, D. Gerbaudo, A. Gershon, H. Ghazlane, N. Ghodbane, B. Giacobbe, S. Giagu, V. Giangiobbe, P. Giannetti, F. Gianotti, B. Gibbard, S. M. Gibson, M. Gilchriese, T. P. S. Gillam, D. Gillberg, G. Gilles, D. M. Gingrich, N. Giokaris, M. P. Giordani, R. Giordano, F. M. Giorgi, F. M. Giorgi, P. F. Giraud, D. Giugni, C. Giuliani, M. Giulini, B. K. Gjelsten, S. Gkaitatzis, I. Gkialas, L. K. Gladilin, C. Glasman, J. Glatzer, P. C. F. Glaysher, A. Glazov, G. L. Glonti, M. Goblirsch-Kolb, J. R. Goddard, J. Godlewski, C. Goeringer, S. Goldfarb, T. Golling, D. Golubkov, A. Gomes, L. S. Gomez Fajardo, R. Gonçalo, J. Goncalves Pinto Firmino Da Costa, L. Gonella, S. González de la Hoz, G. Gonzalez Parra, S. Gonzalez-Sevilla, L. Goossens, P. A. Gorbounov, H. A. Gordon, I. Gorelov, B. Gorini, E. Gorini, A. Gorišek, E. Gornicki, A. T. Goshaw, C. Gössling, M. I. Gostkin, M. Gouighri, D. Goujdami, M. P. Goulette, A. G. Goussiou, C. Goy, S. Gozpinar, H. M. X. Grabas, L. Graber, I. Grabowska-Bold, P. Grafström, K-J. Grahn, J. Gramling, E. Gramstad, S. Grancagnolo, V. Grassi, V. Gratchev, H. M. Gray, E. Graziani, O. G. Grebenyuk, Z. D. Greenwood, K. Gregersen, I. M. Gregor, P. Grenier, J. Griffiths, A. A. Grillo, K. Grimm, S. Grinstein, Ph. Gris, Y. V. Grishkevich, J.-F. Grivaz, J. P. Grohs, A. Grohsjean, E. Gross, J. Grosse-Knetter, G. C. Grossi, J. Groth-Jensen, Z. J. Grout, L. Guan, F. Guescini, D. Guest, O. Gueta, C. Guicheney, E. Guido, T. Guillemin, S. Guindon, U. Gul, C. Gumpert, J. Gunther, J. Guo, S. Gupta, P. Gutierrez, N. G. Gutierrez Ortiz, C. Gutschow, N. Guttman, C. Guyot, C. Gwenlan, C. B. Gwilliam, A. Haas, C. Haber, H. K. Hadavand, N. Haddad, P. Haefner, S. Hageböeck, Z. Hajduk, H. Hakobyan, M. Haleem, D. Hall, G. Halladjian, K. Hamacher, P. Hamal, K. Hamano, M. Hamer, A. Hamilton, S. Hamilton, G. N. Hamity, P. G. Hamnett, L. Han, K. Hanagaki, K. Hanawa, M. Hance, P. Hanke, R. Hanna, J. B. Hansen, J. D. Hansen, P. H. Hansen, K. Hara, A. S. Hard, T. Harenberg, F. Hariri, S. Harkusha, D. Harper, R. D. Harrington, O. M. Harris, P. F. Harrison, F. Hartjes, M. Hasegawa, S. Hasegawa, Y. Hasegawa, A. Hasib, S. Hassani, S. Haug, M. Hauschild, R. Hauser, M. Havranek, C. M. Hawkes, R. J. Hawkings, A. D. Hawkins, T. Hayashi, D. Hayden, C. P. Hays, H. S. Hayward, S. J. Haywood, S. J. Head, T. Heck, V. Hedberg, L. Heelan, S. Heim, T. Heim, B. Heinemann, L. Heinrich, J. Hejbal, L. Helary, C. Heller, M. Heller, S. Hellman, D. Hellmich, C. Helsens, J. Henderson, R. C. W. Henderson, Y. Heng, C. Hengler, A. Henrichs, A. M. Henriques Correia, S. Henrot-Versille, C. Hensel, G. H. Herbert, Y. Hernández Jiménez, R. Herrberg-Schubert, G. Herten, R. Hertenberger, L. Hervas, G. G. Hesketh, N. P. Hessey, R. Hickling, E. Higón-Rodriguez, E. Hill, J. C. Hill, K. H. Hiller, S. Hillert, S. J. Hillier, I. Hinchliffe, E. Hines, M. Hirose, D. Hirschbuehl, J. Hobbs, N. Hod, M. C. Hodgkinson, P. Hodgson, A. Hoecker, M. R. Hoeferkamp, F. Hoenig, J. Hoffman, D. Hoffmann, J. I. Hofmann, M. Hohlfeld, T. R. Holmes, T. M. Hong, L. Hooft van Huysduynen, W. H. Hopkins, Y. Horii, J-Y. Hostachy, S. Hou, A. Hoummada, J. Howard, J. Howarth, M. Hrabovsky, I. Hristova, J. Hrivnac, T. Hryn’ova, C. Hsu, P. J. Hsu, S.-C. Hsu, D. Hu, X. Hu, Y. Huang, Z. Hubacek, F. Hubaut, F. Huegging, T. B. Huffman, E. W. Hughes, G. Hughes, M. Huhtinen, T. A. Hülsing, M. Hurwitz, N. Huseynov, J. Huston, J. Huth, G. Iacobucci, G. Iakovidis, I. Ibragimov, L. Iconomidou-Fayard, E. Ideal, P. Iengo, O. Igonkina, T. Iizawa, Y. Ikegami, K. Ikematsu, M. Ikeno, Y. Ilchenko, D. Iliadis, N. Ilic, Y. Inamaru, T. Ince, P. Ioannou, M. Iodice, K. Iordanidou, V. Ippolito, A. Irles Quiles, C. Isaksson, M. Ishino, M. Ishitsuka, R. Ishmukhametov, C. Issever, S. Istin, J. M. Iturbe Ponce, R. Iuppa, J. Ivarsson, W. Iwanski, H. Iwasaki, J. M. Izen, V. Izzo, B. Jackson, M. Jackson, P. Jackson, M. R. Jaekel, V. Jain, K. Jakobs, S. Jakobsen, T. Jakoubek, J. Jakubek, D. O. Jamin, D. K. Jana, E. Jansen, H. Jansen, J. Janssen, M. Janus, G. Jarlskog, N. Javadov, T. Javůrek, L. Jeanty, J. Jejelava, G.-Y. Jeng, D. Jennens, P. Jenni, J. Jentzsch, C. Jeske, S. Jézéquel, H. Ji, J. Jia, Y. Jiang, M. Jimenez Belenguer, S. Jin, A. Jinaru, O. Jinnouchi, M. D. Joergensen, K. E. Johansson, P. Johansson, K. A. Johns, K. Jon-And, G. Jones, R. W. L. Jones, T. J. Jones, J. Jongmanns, P. M. Jorge, K. D. Joshi, J. Jovicevic, X. Ju, C. A. Jung, R. M. Jungst, P. Jussel, A. Juste Rozas, M. Kaci, A. Kaczmarska, M. Kado, H. Kagan, M. Kagan, E. Kajomovitz, C. W. Kalderon, S. Kama, A. Kamenshchikov, N. Kanaya, M. Kaneda, S. Kaneti, V. A. Kantserov, J. Kanzaki, B. Kaplan, A. Kapliy, D. Kar, K. Karakostas, N. Karastathis, M. J. Kareem, M. Karnevskiy, S. N. Karpov, Z. M. Karpova, K. Karthik, V. Kartvelishvili, A. N. Karyukhin, L. Kashif, G. Kasieczka, R. D. Kass, A. Kastanas, Y. Kataoka, A. Katre, J. Katzy, V. Kaushik, K. Kawagoe, T. Kawamoto, G. Kawamura, S. Kazama, V. F. Kazanin, M. Y. Kazarinov, R. Keeler, R. Kehoe, M. Keil, J. S. Keller, J. J. Kempster, H. Keoshkerian, O. Kepka, B. P. Kerševan, S. Kersten, K. Kessoku, J. Keung, F. Khalil-zada, H. Khandanyan, A. Khanov, A. Khodinov, A. Khomich, T. J. Khoo, G. Khoriauli, A. Khoroshilov, V. Khovanskiy, E. Khramov, J. Khubua, H. Y. Kim, H. Kim, S. H. Kim, N. Kimura, O. Kind, B. T. King, M. King, R. S. B. King, S. B. King, J. Kirk, A. E. Kiryunin, T. Kishimoto, D. Kisielewska, F. Kiss, T. Kittelmann, K. Kiuchi, E. Kladiva, M. Klein, U. Klein, K. Kleinknecht, P. Klimek, A. Klimentov, R. Klingenberg, J. A. Klinger, T. Klioutchnikova, P. F. Klok, E.-E. Kluge, P. Kluit, S. Kluth, E. Kneringer, E. B. F. G. Knoops, A. Knue, D. Kobayashi, T. Kobayashi, M. Kobel, M. Kocian, P. Kodys, P. Koevesarki, T. Koffas, E. Koffeman, L. A. Kogan, S. Kohlmann, Z. Kohout, T. Kohriki, T. Koi, H. Kolanoski, I. Koletsou, J. Koll, A. A. Komar, Y. Komori, T. Kondo, N. Kondrashova, K. Köneke, A. C. König, S. König, T. Kono, R. Konoplich, N. Konstantinidis, R. Kopeliansky, S. Koperny, L. Köpke, A. K. Kopp, K. Korcyl, K. Kordas, A. Korn, A. A. Korol, I. Korolkov, E. V. Korolkova, V. A. Korotkov, O. Kortner, S. Kortner, V. V. Kostyukhin, V. M. Kotov, A. Kotwal, C. Kourkoumelis, V. Kouskoura, A. Koutsman, R. Kowalewski, T. Z. Kowalski, W. Kozanecki, A. S. Kozhin, V. Kral, V. A. Kramarenko, G. Kramberger, D. Krasnopevtsev, M. W. Krasny, A. Krasznahorkay, J. K. Kraus, A. Kravchenko, S. Kreiss, M. Kretz, J. Kretzschmar, K. Kreutzfeldt, P. Krieger, K. Kroeninger, H. Kroha, J. Kroll, J. Kroseberg, J. Krstic, U. Kruchonak, H. Krüger, T. Kruker, N. Krumnack, Z. V. Krumshteyn, A. Kruse, M. C. Kruse, M. Kruskal, T. Kubota, S. Kuday, S. Kuehn, A. Kugel, A. Kuhl, T. Kuhl, V. Kukhtin, Y. Kulchitsky, S. Kuleshov, M. Kuna, J. Kunkle, A. Kupco, H. Kurashige, Y. A. Kurochkin, R. Kurumida, V. Kus, E. S. Kuwertz, M. Kuze, J. Kvita, A. La Rosa, L. La Rotonda, C. Lacasta, F. Lacava, J. Lacey, H. Lacker, D. Lacour, V. R. Lacuesta, E. Ladygin, R. Lafaye, B. Laforge, T. Lagouri, S. Lai, H. Laier, L. Lambourne, S. Lammers, C. L. Lampen, W. Lampl, E. Lançon, U. Landgraf, M. P. J. Landon, V. S. Lang, A. J. Lankford, F. Lanni, K. Lantzsch, S. Laplace, C. Lapoire, J. F. Laporte, T. Lari, F. Lasagni Manghi, M. Lassnig, P. Laurelli, W. Lavrijsen, A. T. Law, P. Laycock, O. Le Dortz, E. Le Guirriec, E. Le Menedeu, T. LeCompte, F. Ledroit-Guillon, C. A. Lee, H. Lee, J. S. H. Lee, S. C. Lee, L. Lee, G. Lefebvre, M. Lefebvre, F. Legger, C. Leggett, A. Lehan, M. Lehmacher, G. Lehmann Miotto, X. Lei, W. A. Leight, A. Leisos, A. G. Leister, M. A. L. Leite, R. Leitner, D. Lellouch, B. Lemmer, K. J. C. Leney, T. Lenz, G. Lenzen, B. Lenzi, R. Leone, S. Leone, C. Leonidopoulos, S. Leontsinis, C. Leroy, C. G. Lester, C. M. Lester, M. Levchenko, J. Levêque, D. Levin, L. J. Levinson, M. Levy, A. Lewis, G. H. Lewis, A. M. Leyko, M. Leyton, B. Li, B. Li, H. Li, H. L. Li, L. Li, L. Li, S. Li, Y. Li, Z. Liang, H. Liao, B. Liberti, P. Lichard, K. Lie, J. Liebal, W. Liebig, C. Limbach, A. Limosani, S. C. Lin, T. H. Lin, F. Linde, B. E. Lindquist, J. T. Linnemann, E. Lipeles, A. Lipniacka, M. Lisovyi, T. M. Liss, D. Lissauer, A. Lister, A. M. Litke, B. Liu, D. Liu, J. B. Liu, K. Liu, L. Liu, M. Liu, M. Liu, Y. Liu, M. Livan, S. S. A. Livermore, A. Lleres, J. Llorente Merino, S. L. Lloyd, F. Lo Sterzo, E. Lobodzinska, P. Loch, W. S. Lockman, T. Loddenkoetter, F. K. Loebinger, A. E. Loevschall-Jensen, A. Loginov, T. Lohse, K. Lohwasser, M. Lokajicek, V. P. Lombardo, B. A. Long, J. D. Long, R. E. Long, L. Lopes, D. Lopez Mateos, B. Lopez Paredes, I. Lopez Paz, J. Lorenz, N. Lorenzo Martinez, M. Losada, P. Loscutoff, X. Lou, A. Lounis, J. Love, P. A. Love, A. J. Lowe, F. Lu, N. Lu, H. J. Lubatti, C. Luci, A. Lucotte, F. Luehring, W. Lukas, L. Luminari, O. Lundberg, B. Lund-Jensen, M. Lungwitz, D. Lynn, R. Lysak, E. Lytken, H. Ma, L. L. Ma, G. Maccarrone, A. Macchiolo, J. Machado Miguens, D. Macina, D. Madaffari, R. Madar, H. J. Maddocks, W. F. Mader, A. Madsen, M. Maeno, T. Maeno, A. Maevskiy, E. Magradze, K. Mahboubi, J. Mahlstedt, S. Mahmoud, C. Maiani, C. Maidantchik, A. A. Maier, A. Maio, S. Majewski, Y. Makida, N. Makovec, P. Mal, B. Malaescu, Pa. Malecki, V. P. Maleev, F. Malek, U. Mallik, D. Malon, C. Malone, S. Maltezos, V. M. Malyshev, S. Malyukov, J. Mamuzic, B. Mandelli, L. Mandelli, I. Mandić, R. Mandrysch, J. Maneira, A. Manfredini, L. Manhaes de Andrade Filho, J. A. Manjarres Ramos, A. Mann, P. M. Manning, A. Manousakis-Katsikakis, B. Mansoulie, R. Mantifel, L. Mapelli, L. March, J. F. Marchand, G. Marchiori, M. Marcisovsky, C. P. Marino, M. Marjanovic, C. N. Marques, F. Marroquim, S. P. Marsden, Z. Marshall, L. F. Marti, S. Marti-Garcia, B. Martin, B. Martin, T. A. Martin, V. J. Martin, B. Martin dit Latour, H. Martinez, M. Martinez, S. Martin-Haugh, A. C. Martyniuk, M. Marx, F. Marzano, A. Marzin, L. Masetti, T. Mashimo, R. Mashinistov, J. Masik, A. L. Maslennikov, I. Massa, L. Massa, N. Massol, P. Mastrandrea, A. Mastroberardino, T. Masubuchi, P. Mättig, J. Mattmann, J. Maurer, S. J. Maxfield, D. A. Maximov, R. Mazini, L. Mazzaferro, G. Mc Goldrick, S. P. Mc Kee, A. McCarn, R. L. McCarthy, T. G. McCarthy, N. A. McCubbin, K. W. McFarlane, J. A. Mcfayden, G. Mchedlidze, S. J. McMahon, R. A. McPherson, J. Mechnich, M. Medinnis, S. Meehan, S. Mehlhase, A. Mehta, K. Meier, C. Meineck, B. Meirose, C. Melachrinos, B. R. Mellado Garcia, F. Meloni, A. Mengarelli, S. Menke, E. Meoni, K. M. Mercurio, S. Mergelmeyer, N. Meric, P. Mermod, L. Merola, C. Meroni, F. S. Merritt, H. Merritt, A. Messina, J. Metcalfe, A. S. Mete, C. Meyer, C. Meyer, J-P. Meyer, J. Meyer, R. P. Middleton, S. Migas, L. Mijović, G. Mikenberg, M. Mikestikova, M. Mikuž, A. Milic, D. W. Miller, C. Mills, A. Milov, D. A. Milstead, D. Milstein, A. A. Minaenko, I. A. Minashvili, A. I. Mincer, B. Mindur, M. Mineev, Y. Ming, L. M. Mir, G. Mirabelli, T. Mitani, J. Mitrevski, V. A. Mitsou, S. Mitsui, A. Miucci, P. S. Miyagawa, J. U. Mjörnmark, T. Moa, K. Mochizuki, S. Mohapatra, W. Mohr, S. Molander, R. Moles-Valls, K. Mönig, C. Monini, J. Monk, E. Monnier, J. Montejo Berlingen, F. Monticelli, S. Monzani, R. W. Moore, N. Morange, D. Moreno, M. Moreno Llácer, P. Morettini, M. Morgenstern, M. Morii, S. Moritz, A. K. Morley, G. Mornacchi, J. D. Morris, L. Morvaj, H. G. Moser, M. Mosidze, J. Moss, K. Motohashi, R. Mount, E. Mountricha, S. V. Mouraviev, E. J. W. Moyse, S. Muanza, R. D. Mudd, F. Mueller, J. Mueller, K. Mueller, T. Mueller, T. Mueller, D. Muenstermann, Y. Munwes, J. A. Murillo Quijada, W. J. Murray, H. Musheghyan, E. Musto, A. G. Myagkov, M. Myska, O. Nackenhorst, J. Nadal, K. Nagai, R. Nagai, Y. Nagai, K. Nagano, A. Nagarkar, Y. Nagasaka, M. Nagel, A. M. Nairz, Y. Nakahama, K. Nakamura, T. Nakamura, I. Nakano, H. Namasivayam, G. Nanava, R. Narayan, T. Nattermann, T. Naumann, G. Navarro, R. Nayyar, H. A. Neal, P. Yu. Nechaeva, T. J. Neep, P. D. Nef, A. Negri, G. Negri, M. Negrini, S. Nektarijevic, C. Nellist, A. Nelson, T. K. Nelson, S. Nemecek, P. Nemethy, A. A. Nepomuceno, M. Nessi, M. S. Neubauer, M. Neumann, R. M. Neves, P. Nevski, P. R. Newman, D. H. Nguyen, R. B. Nickerson, R. Nicolaidou, B. Nicquevert, J. Nielsen, N. Nikiforou, A. Nikiforov, V. Nikolaenko, I. Nikolic-Audit, K. Nikolics, K. Nikolopoulos, P. Nilsson, Y. Ninomiya, A. Nisati, R. Nisius, T. Nobe, L. Nodulman, M. Nomachi, I. Nomidis, S. Norberg, M. Nordberg, O. Novgorodova, S. Nowak, M. Nozaki, L. Nozka, K. Ntekas, G. Nunes Hanninger, T. Nunnemann, E. Nurse, F. Nuti, B. J. O’Brien, F. O’grady, D. C. O’Neil, V. O’Shea, F. G. Oakham, H. Oberlack, T. Obermann, J. Ocariz, A. Ochi, M. I. Ochoa, S. Oda, S. Odaka, H. Ogren, A. Oh, S. H. Oh, C. C. Ohm, H. Ohman, W. Okamura, H. Okawa, Y. Okumura, T. Okuyama, A. Olariu, A. G. Olchevski, S. A. Olivares Pino, D. Oliveira Damazio, E. Oliver Garcia, A. Olszewski, J. Olszowska, A. Onofre, P. U. E. Onyisi, C. J. Oram, M. J. Oreglia, Y. Oren, D. Orestano, N. Orlando, C. Oropeza Barrera, R. S. Orr, B. Osculati, R. Ospanov, G. Otero y Garzon, H. Otono, M. Ouchrif, E. A. Ouellette, F. Ould-Saada, A. Ouraou, K. P. Oussoren, Q. Ouyang, A. Ovcharova, M. Owen, V. E. Ozcan, N. Ozturk, K. Pachal, A. Pacheco Pages, C. Padilla Aranda, M. Pagáčová, S. Pagan Griso, E. Paganis, C. Pahl, F. Paige, P. Pais, K. Pajchel, G. Palacino, S. Palestini, M. Palka, D. Pallin, A. Palma, J. D. Palmer, Y. B. Pan, E. Panagiotopoulou, J. G. Panduro Vazquez, P. Pani, N. Panikashvili, S. Panitkin, D. Pantea, L. Paolozzi, Th. D. Papadopoulou, K. Papageorgiou, A. Paramonov, D. Paredes Hernandez, M. A. Parker, F. Parodi, J. A. Parsons, U. Parzefall, E. Pasqualucci, S. Passaggio, A. Passeri, F. Pastore, Fr. Pastore, G. Pásztor, S. Pataraia, N. D. Patel, J. R. Pater, S. Patricelli, T. Pauly, J. Pearce, L. E. Pedersen, M. Pedersen, S. Pedraza Lopez, R. Pedro, S. V. Peleganchuk, D. Pelikan, H. Peng, B. Penning, J. Penwell, D. V. Perepelitsa, E. Perez Codina, M. T. Pérez García-Estañ, V. Perez Reale, L. Perini, H. Pernegger, S. Perrella, R. Perrino, R. Peschke, V. D. Peshekhonov, K. Peters, R. F. Y. Peters, B. A. Petersen, T. C. Petersen, E. Petit, A. Petridis, C. Petridou, E. Petrolo, F. Petrucci, N. E. Pettersson, R. Pezoa, P. W. Phillips, G. Piacquadio, E. Pianori, A. Picazio, E. Piccaro, M. Piccinini, R. Piegaia, D. T. Pignotti, J. E. Pilcher, A. D. Pilkington, J. Pina, M. Pinamonti, A. Pinder, J. L. Pinfold, A. Pingel, B. Pinto, S. Pires, M. Pitt, C. Pizio, L. Plazak, M.-A. Pleier, V. Pleskot, E. Plotnikova, P. Plucinski, S. Poddar, F. Podlyski, R. Poettgen, L. Poggioli, D. Pohl, M. Pohl, G. Polesello, A. Policicchio, R. Polifka, A. Polini, C. S. Pollard, V. Polychronakos, K. Pommès, L. Pontecorvo, B. G. Pope, G. A. Popeneciu, D. S. Popovic, A. Poppleton, X. Portell Bueso, S. Pospisil, K. Potamianos, I. N. Potrap, C. J. Potter, C. T. Potter, G. Poulard, J. Poveda, V. Pozdnyakov, P. Pralavorio, A. Pranko, S. Prasad, R. Pravahan, S. Prell, D. Price, J. Price, L. E. Price, D. Prieur, M. Primavera, M. Proissl, K. Prokofiev, F. Prokoshin, E. Protopapadaki, S. Protopopescu, J. Proudfoot, M. Przybycien, H. Przysiezniak, E. Ptacek, D. Puddu, E. Pueschel, D. Puldon, M. Purohit, P. Puzo, J. Qian, G. Qin, Y. Qin, A. Quadt, D. R. Quarrie, W. B. Quayle, M. Queitsch-Maitland, D. Quilty, A. Qureshi, V. Radeka, V. Radescu, S. K. Radhakrishnan, P. Radloff, P. Rados, F. Ragusa, G. Rahal, S. Rajagopalan, M. Rammensee, A. S. Randle-Conde, C. Rangel-Smith, K. Rao, F. Rauscher, T. C. Rave, T. Ravenscroft, M. Raymond, A. L. Read, N. P. Readioff, D. M. Rebuzzi, A. Redelbach, G. Redlinger, R. Reece, K. Reeves, L. Rehnisch, H. Reisin, M. Relich, C. Rembser, H. Ren, Z. L. Ren, A. Renaud, M. Rescigno, S. Resconi, O. L. Rezanova, P. Reznicek, R. Rezvani, R. Richter, M. Ridel, P. Rieck, J. Rieger, M. Rijssenbeek, A. Rimoldi, L. Rinaldi, E. Ritsch, I. Riu, F. Rizatdinova, E. Rizvi, S. H. Robertson, A. Robichaud-Veronneau, D. Robinson, J. E. M. Robinson, A. Robson, C. Roda, L. Rodrigues, S. Roe, O. Røhne, S. Rolli, A. Romaniouk, M. Romano, E. Romero Adam, N. Rompotis, M. Ronzani, L. Roos, E. Ros, S. Rosati, K. Rosbach, M. Rose, P. Rose, P. L. Rosendahl, O. Rosenthal, V. Rossetti, E. Rossi, L. P. Rossi, R. Rosten, M. Rotaru, I. Roth, J. Rothberg, D. Rousseau, C. R. Royon, A. Rozanov, Y. Rozen, X. Ruan, F. Rubbo, I. Rubinskiy, V. I. Rud, C. Rudolph, M. S. Rudolph, F. Rühr, A. Ruiz-Martinez, Z. Rurikova, N. A. Rusakovich, A. Ruschke, J. P. Rutherfoord, N. Ruthmann, Y. F. Ryabov, M. Rybar, G. Rybkin, N. C. Ryder, A. F. Saavedra, S. Sacerdoti, A. Saddique, I. Sadeh, H. F-W. Sadrozinski, R. Sadykov, F. Safai Tehrani, H. Sakamoto, Y. Sakurai, G. Salamanna, A. Salamon, M. Saleem, D. Salek, P. H. Sales De Bruin, D. Salihagic, A. Salnikov, J. Salt, D. Salvatore, F. Salvatore, A. Salvucci, A. Salzburger, D. Sampsonidis, A. Sanchez, J. Sánchez, V. Sanchez Martinez, H. Sandaker, R. L. Sandbach, H. G. Sander, M. P. Sanders, M. Sandhoff, T. Sandoval, C. Sandoval, R. Sandstroem, D. P. C. Sankey, A. Sansoni, C. Santoni, R. Santonico, H. Santos, I. Santoyo Castillo, K. Sapp, A. Sapronov, J. G. Saraiva, B. Sarrazin, G. Sartisohn, O. Sasaki, Y. Sasaki, G. Sauvage, E. Sauvan, P. Savard, D. O. Savu, C. Sawyer, L. Sawyer, D. H. Saxon, J. Saxon, C. Sbarra, A. Sbrizzi, T. Scanlon, D. A. Scannicchio, M. Scarcella, V. Scarfone, J. Schaarschmidt, P. Schacht, D. Schaefer, R. Schaefer, S. Schaepe, S. Schaetzel, U. Schäfer, A. C. Schaffer, D. Schaile, R. D. Schamberger, V. Scharf, V. A. Schegelsky, D. Scheirich, M. Schernau, M. I. Scherzer, C. Schiavi, J. Schieck, C. Schillo, M. Schioppa, S. Schlenker, E. Schmidt, K. Schmieden, C. Schmitt, S. Schmitt, B. Schneider, Y. J. Schnellbach, U. Schnoor, L. Schoeffel, A. Schoening, B. D. Schoenrock, A. L. S. Schorlemmer, M. Schott, D. Schouten, J. Schovancova, S. Schramm, M. Schreyer, C. Schroeder, N. Schuh, M. J. Schultens, H.-C. Schultz-Coulon, H. Schulz, M. Schumacher, B. A. Schumm, Ph. Schune, C. Schwanenberger, A. Schwartzman, T. A. Schwarz, Ph. Schwegler, Ph. Schwemling, R. Schwienhorst, J. Schwindling, T. Schwindt, M. Schwoerer, F. G. Sciacca, E. Scifo, G. Sciolla, W. G. Scott, F. Scuri, F. Scutti, J. Searcy, G. Sedov, E. Sedykh, S. C. Seidel, A. Seiden, F. Seifert, J. M. Seixas, G. Sekhniaidze, S. J. Sekula, K. E. Selbach, D. M. Seliverstov, G. Sellers, N. Semprini-Cesari, C. Serfon, L. Serin, L. Serkin, T. Serre, R. Seuster, H. Severini, T. Sfiligoj, F. Sforza, A. Sfyrla, E. Shabalina, M. Shamim, L. Y. Shan, R. Shang, J. T. Shank, M. Shapiro, P. B. Shatalov, K. Shaw, C. Y. Shehu, P. Sherwood, L. Shi, S. Shimizu, C. O. Shimmin, M. Shimojima, M. Shiyakova, A. Shmeleva, M. J. Shochet, D. Short, S. Shrestha, E. Shulga, M. A. Shupe, S. Shushkevich, P. Sicho, O. Sidiropoulou, D. Sidorov, A. Sidoti, F. Siegert, Dj. Sijacki, J. Silva, Y. Silver, D. Silverstein, S. B. Silverstein, V. Simak, O. Simard, Lj. Simic, S. Simion, E. Simioni, B. Simmons, R. Simoniello, M. Simonyan, P. Sinervo, N. B. Sinev, V. Sipica, G. Siragusa, A. Sircar, A. N. Sisakyan, S. Yu. Sivoklokov, J. Sjölin, T. B. Sjursen, H. P. Skottowe, K. Yu. Skovpen, P. Skubic, M. Slater, T. Slavicek, K. Sliwa, V. Smakhtin, B. H. Smart, L. Smestad, S. Yu. Smirnov, Y. Smirnov, L. N. Smirnova, O. Smirnova, K. M. Smith, M. Smizanska, K. Smolek, A. A. Snesarev, G. Snidero, S. Snyder, R. Sobie, F. Socher, A. Soffer, D. A. Soh, C. A. Solans, M. Solar, J. Solc, E. Yu. Soldatov, U. Soldevila, A. A. Solodkov, A. Soloshenko, O. V. Solovyanov, V. Solovyev, P. Sommer, H. Y. Song, N. Soni, A. Sood, A. Sopczak, B. Sopko, V. Sopko, V. Sorin, M. Sosebee, R. Soualah, P. Soueid, A. M. Soukharev, D. South, S. Spagnolo, F. Spanò, W. R. Spearman, F. Spettel, R. Spighi, G. Spigo, L. A. Spiller, M. Spousta, T. Spreitzer, B. Spurlock, R. D. St. Denis, S. Staerz, J. Stahlman, R. Stamen, S. Stamm, E. Stanecka, R. W. Stanek, C. Stanescu, M. Stanescu-Bellu, M. M. Stanitzki, S. Stapnes, E. A. Starchenko, J. Stark, P. Staroba, P. Starovoitov, R. Staszewski, P. Stavina, P. Steinberg, B. Stelzer, H. J. Stelzer, O. Stelzer-Chilton, H. Stenzel, S. Stern, G. A. Stewart, J. A. Stillings, M. C. Stockton, M. Stoebe, G. Stoicea, P. Stolte, S. Stonjek, A. R. Stradling, A. Straessner, M. E. Stramaglia, J. Strandberg, S. Strandberg, A. Strandlie, E. Strauss, M. Strauss, P. Strizenec, R. Ströhmer, D. M. Strom, R. Stroynowski, A. Strubig, S. A. Stucci, B. Stugu, N. A. Styles, D. Su, J. Su, R. Subramaniam, A. Succurro, Y. Sugaya, C. Suhr, M. Suk, V. V. Sulin, S. Sultansoy, T. Sumida, S. Sun, X. Sun, J. E. Sundermann, K. Suruliz, G. Susinno, M. R. Sutton, Y. Suzuki, M. Svatos, S. Swedish, M. Swiatlowski, I. Sykora, T. Sykora, D. Ta, C. Taccini, K. Tackmann, J. Taenzer, A. Taffard, R. Tafirout, N. Taiblum, H. Takai, R. Takashima, H. Takeda, T. Takeshita, Y. Takubo, M. Talby, A. A. Talyshev, J. Y. C. Tam, K. G. Tan, J. Tanaka, R. Tanaka, S. Tanaka, S. Tanaka, A. J. Tanasijczuk, B. B. Tannenwald, N. Tannoury, S. Tapprogge, S. Tarem, F. Tarrade, G. F. Tartarelli, P. Tas, M. Tasevsky, T. Tashiro, E. Tassi, A. Tavares Delgado, Y. Tayalati, F. E. Taylor, G. N. Taylor, W. Taylor, F. A. Teischinger, M. Teixeira Dias Castanheira, P. Teixeira-Dias, K. K. Temming, H. Ten Kate, P. K. Teng, J. J. Teoh, S. Terada, K. Terashi, J. Terron, S. Terzo, M. Testa, R. J. Teuscher, J. Therhaag, T. Theveneaux-Pelzer, J. P. Thomas, J. Thomas-Wilsker, E. N. Thompson, P. D. Thompson, P. D. Thompson, R. J. Thompson, A. S. Thompson, L. A. Thomsen, E. Thomson, M. Thomson, W. M. Thong, R. P. Thun, F. Tian, M. J. Tibbetts, V. O. Tikhomirov, Yu. A. Tikhonov, S. Timoshenko, E. Tiouchichine, P. Tipton, S. Tisserant, T. Todorov, S. Todorova-Nova, B. Toggerson, J. Tojo, S. Tokár, K. Tokushuku, K. Tollefson, E. Tolley, L. Tomlinson, M. Tomoto, L. Tompkins, K. Toms, N. D. Topilin, E. Torrence, H. Torres, E. Torró Pastor, J. Toth, F. Touchard, D. R. Tovey, H. L. Tran, T. Trefzger, L. Tremblet, A. Tricoli, I. M. Trigger, S. Trincaz-Duvoid, M. F. Tripiana, W. Trischuk, B. Trocmé, C. Troncon, M. Trottier-McDonald, M. Trovatelli, P. True, M. Trzebinski, A. Trzupek, C. Tsarouchas, J. C-L. Tseng, P. V. Tsiareshka, D. Tsionou, G. Tsipolitis, N. Tsirintanis, S. Tsiskaridze, V. Tsiskaridze, E. G. Tskhadadze, I. I. Tsukerman, V. Tsulaia, S. Tsuno, D. Tsybychev, A. Tudorache, V. Tudorache, A. N. Tuna, S. A. Tupputi, S. Turchikhin, D. Turecek, I. Turk Cakir, R. Turra, P. M. Tuts, A. Tykhonov, M. Tylmad, M. Tyndel, K. Uchida, I. Ueda, R. Ueno, M. Ughetto, M. Ugland, M. Uhlenbrock, F. Ukegawa, G. Unal, A. Undrus, G. Unel, F. C. Ungaro, Y. Unno, C. Unverdorben, D. Urbaniec, P. Urquijo, G. Usai, A. Usanova, L. Vacavant, V. Vacek, B. Vachon, N. Valencic, S. Valentinetti, A. Valero, L. Valery, S. Valkar, E. Valladolid Gallego, S. Vallecorsa, J. A. Valls Ferrer, W. Van Den Wollenberg, P. C. Van Der Deijl, R. van der Geer, H. van der Graaf, R. Van Der Leeuw, D. van der Ster, N. van Eldik, P. van Gemmeren, J. Van Nieuwkoop, I. van Vulpen, M. C. van Woerden, M. Vanadia, W. Vandelli, R. Vanguri, A. Vaniachine, P. Vankov, F. Vannucci, G. Vardanyan, R. Vari, E. W. Varnes, T. Varol, D. Varouchas, A. Vartapetian, K. E. Varvell, F. Vazeille, T. Vazquez Schroeder, J. Veatch, F. Veloso, S. Veneziano, A. Ventura, D. Ventura, M. Venturi, N. Venturi, A. Venturini, V. Vercesi, M. Verducci, W. Verkerke, J. C. Vermeulen, A. Vest, M. C. Vetterli, O. Viazlo, I. Vichou, T. Vickey, O. E. Vickey Boeriu, G. H. A. Viehhauser, S. Viel, R. Vigne, M. Villa, M. Villaplana Perez, E. Vilucchi, M. G. Vincter, V. B. Vinogradov, J. Virzi, I. Vivarelli, F. Vives Vaque, S. Vlachos, D. Vladoiu, M. Vlasak, A. Vogel, M. Vogel, P. Vokac, G. Volpi, M. Volpi, H. von der Schmitt, H. von Radziewski, E. von Toerne, V. Vorobel, K. Vorobev, M. Vos, R. Voss, J. H. Vossebeld, N. Vranjes, M. Vranjes Milosavljevic, V. Vrba, M. Vreeswijk, T. Vu Anh, R. Vuillermet, I. Vukotic, Z. Vykydal, P. Wagner, W. Wagner, H. Wahlberg, S. Wahrmund, J. Wakabayashi, J. Walder, R. Walker, W. Walkowiak, R. Wall, P. Waller, B. Walsh, C. Wang, C. Wang, F. Wang, H. Wang, H. Wang, J. Wang, J. Wang, K. Wang, R. Wang, S. M. Wang, T. Wang, X. Wang, C. Wanotayaroj, A. Warburton, C. P. Ward, D. R. Wardrope, M. Warsinsky, A. Washbrook, C. Wasicki, P. M. Watkins, A. T. Watson, I. J. Watson, M. F. Watson, G. Watts, S. Watts, B. M. Waugh, S. Webb, M. S. Weber, S. W. Weber, J. S. Webster, A. R. Weidberg, P. Weigell, B. Weinert, J. Weingarten, C. Weiser, H. Weits, P. S. Wells, T. Wenaus, D. Wendland, Z. Weng, T. Wengler, S. Wenig, N. Wermes, M. Werner, P. Werner, M. Wessels, J. Wetter, K. Whalen, A. White, M. J. White, R. White, S. White, D. Whiteson, D. Wicke, F. J. Wickens, W. Wiedenmann, M. Wielers, P. Wienemann, C. Wiglesworth, L. A. M. Wiik-Fuchs, P. A. Wijeratne, A. Wildauer, M. A. Wildt, H. G. Wilkens, J. Z. Will, H. H. Williams, S. Williams, C. Willis, S. Willocq, A. Wilson, J. A. Wilson, I. Wingerter-Seez, F. Winklmeier, B. T. Winter, M. Wittgen, T. Wittig, J. Wittkowski, S. J. Wollstadt, M. W. Wolter, H. Wolters, B. K. Wosiek, J. Wotschack, M. J. Woudstra, K. W. Wozniak, M. Wright, M. Wu, S. L. Wu, X. Wu, Y. Wu, E. Wulf, T. R. Wyatt, B. M. Wynne, S. Xella, M. Xiao, D. Xu, L. Xu, B. Yabsley, S. Yacoob, R. Yakabe, M. Yamada, H. Yamaguchi, Y. Yamaguchi, A. Yamamoto, K. Yamamoto, S. Yamamoto, T. Yamamura, T. Yamanaka, K. Yamauchi, Y. Yamazaki, Z. Yan, H. Yang, H. Yang, U. K. Yang, Y. Yang, S. Yanush, L. Yao, W-M. Yao, Y. Yasu, E. Yatsenko, K. H. Yau Wong, J. Ye, S. Ye, I. Yeletskikh, A. L. Yen, E. Yildirim, M. Yilmaz, R. Yoosoofmiya, K. Yorita, R. Yoshida, K. Yoshihara, C. Young, C. J. S. Young, S. Youssef, D. R. Yu, J. Yu, J. M. Yu, J. Yu, L. Yuan, A. Yurkewicz, I. Yusuff, B. Zabinski, R. Zaidan, A. M. Zaitsev, A. Zaman, S. Zambito, L. Zanello, D. Zanzi, C. Zeitnitz, M. Zeman, A. Zemla, K. Zengel, O. Zenin, T. Ženiš, D. Zerwas, G. Zevi della Porta, D. Zhang, F. Zhang, H. Zhang, J. Zhang, L. Zhang, X. Zhang, Z. Zhang, Z. Zhao, A. Zhemchugov, J. Zhong, B. Zhou, L. Zhou, N. Zhou, C. G. Zhu, H. Zhu, J. Zhu, Y. Zhu, X. Zhuang, K. Zhukov, A. Zibell, D. Zieminska, N. I. Zimine, C. Zimmermann, R. Zimmermann, S. Zimmermann, S. Zimmermann, Z. Zinonos, M. Ziolkowski, G. Zobernig, A. Zoccoli, M. zur Nedden, G. Zurzolo, V. Zutshi, L. Zwalinski

**Affiliations:** 1 Department of Physics, University of Adelaide, Adelaide, Australia; 2 Physics Department, SUNY Albany, Albany, NY USA; 3 Department of Physics, University of Alberta, Edmonton, AB Canada; 4 Department of Physics, Ankara University, Ankara, Turkey; Department of Physics, Gazi University, Ankara, Turkey; Division of Physics, TOBB University of Economics and Technology, Ankara, Turkey; Turkish Atomic Energy Authority, Ankara, Turkey; 5 LAPP, CNRS/IN2P3, Université de Savoie, Annecy-le-Vieux, France; 6 High Energy Physics Division, Argonne National Laboratory, Argonne, IL USA; 7 Department of Physics, University of Arizona, Tucson, AZ USA; 8 Department of Physics, The University of Texas at Arlington, Arlington, TX USA; 9 Physics Department, University of Athens, Athens, Greece; 10 Physics Department, National Technical University of Athens, Zografou, Greece; 11 Institute of Physics, Azerbaijan Academy of Sciences, Baku, Azerbaijan; 12 Institut de Física d’Altes Energies and Departament de Física de la Universitat Autònoma de Barcelona, Barcelona, Spain; 13 Institute of Physics, University of Belgrade, Belgrade, Serbia; Vinca Institute of Nuclear Sciences, University of Belgrade, Belgrade, Serbia; 14 Department for Physics and Technology, University of Bergen, Bergen, Norway; 15 Physics Division, Lawrence Berkeley National Laboratory, University of California, Berkeley, CA USA; 16 Department of Physics, Humboldt University, Berlin, Germany; 17 Albert Einstein Center for Fundamental Physics and Laboratory for High Energy Physics, University of Bern, Bern, Switzerland; 18 School of Physics and Astronomy, University of Birmingham, Birmingham, UK; 19 Department of Physics, Bogazici University, Istanbul, Turkey; Department of Physics, Dogus University, Istanbul, Turkey; Department of Physics Engineering, Gaziantep University, Gaziantep, Turkey; 20 INFN Sezione di Bologna, Bologna, Italy; Dipartimento di Fisica e Astronomia, Università di Bologna, Bologna, Italy; 21 Physikalisches Institut, University of Bonn, Bonn, Germany; 22 Department of Physics, Boston University, Boston, MA USA; 23 Department of Physics, Brandeis University, Waltham, MA USA; 24 Universidade Federal do Rio De Janeiro COPPE/EE/IF, Rio de Janeiro, Brazil; Federal University of Juiz de Fora (UFJF), Juiz de Fora, Brazil; Federal University of Sao Joao del Rei (UFSJ), Sao Joao del Rei, Brazil; Instituto de Fisica, Universidade de Sao Paulo, São Paulo, Brazil; 25 Physics Department, Brookhaven National Laboratory, Upton, NY USA; 26 National Institute of Physics and Nuclear Engineering, Bucharest, Romania; Physics Department, National Institute for Research and Development of Isotopic and Molecular Technologies, Cluj Napoca, Romania; University Politehnica Bucharest, Bucharest, Romania; West University in Timisoara, Timisoara, Romania; 27 Departamento de Física, Universidad de Buenos Aires, Buenos Aires, Argentina; 28 Cavendish Laboratory, University of Cambridge, Cambridge, UK; 29 Department of Physics, Carleton University, Ottawa, ON Canada; 30 CERN, Geneva, Switzerland; 31 Enrico Fermi Institute, University of Chicago, Chicago, IL USA; 32 Departamento de Física, Pontificia Universidad Católica de Chile, Santiago, Chile; Departamento de Física, Universidad Técnica Federico Santa María, Valparaiso, Chile; 33 Institute of High Energy Physics, Chinese Academy of Sciences, Beijing, China; Department of Modern Physics, University of Science and Technology of China, Hefei, Anhui, China; Department of Physics, Nanjing University, Nanjing, Jiangsu, China; School of Physics, Shandong University, Jinan, Shandong, China; Physics Department, Shanghai Jiao Tong University, Shanghai, China; 34 Laboratoire de Physique Corpusculaire, Clermont Université and Université Blaise Pascal and CNRS/IN2P3, Clermont-Ferrand, France; 35 Nevis Laboratory, Columbia University, Irvington, NY USA; 36 Niels Bohr Institute, University of Copenhagen, Copenhagen, Denmark; 37 INFN Gruppo Collegato di Cosenza, Laboratori Nazionali di Frascati, Frascati, Italy; Dipartimento di Fisica, Università della Calabria, Rende, Italy; 38 AGH University of Science and Technology, Faculty of Physics and Applied Computer Science, Kraków, Poland; Marian Smoluchowski Institute of Physics, Jagiellonian University, Kraków, Poland; 39 The Henryk Niewodniczanski Institute of Nuclear Physics, Polish Academy of Sciences, Kraków, Poland; 40 Physics Department, Southern Methodist University, Dallas, TX USA; 41 Physics Department, University of Texas at Dallas, Richardson, TX USA; 42 DESY, Hamburg and Zeuthen, Germany; 43 Institut für Experimentelle Physik IV, Technische Universität Dortmund, Dortmund, Germany; 44 Institut für Kern- und Teilchenphysik, Technische Universität Dresden, Dresden, Germany; 45 Department of Physics, Duke University, Durham, NC USA; 46 SUPA, School of Physics and Astronomy, University of Edinburgh, Edinburgh, UK; 47 INFN Laboratori Nazionali di Frascati, Frascati, Italy; 48 Fakultät für Mathematik und Physik, Albert-Ludwigs-Universität, Freiburg, Germany; 49 Section de Physique, Université de Genève, Geneva, Switzerland; 50 INFN Sezione di Genova, Genoa, Italy; Dipartimento di Fisica, Università di Genova, Genoa, Italy; 51 E. Andronikashvili Institute of Physics, Iv. Javakhishvili Tbilisi State University, Tbilisi, Georgia; High Energy Physics Institute, Tbilisi State University, Tbilisi, Georgia; 52 II Physikalisches Institut, Justus-Liebig-Universität Giessen, Giessen, Germany; 53 SUPA, School of Physics and Astronomy, University of Glasgow, Glasgow, UK; 54 II Physikalisches Institut, Georg-August-Universität, Göttingen, Germany; 55 Laboratoire de Physique Subatomique et de Cosmologie, Université Grenoble-Alpes, CNRS/IN2P3, Grenoble, France; 56 Department of Physics, Hampton University, Hampton, VA USA; 57 Laboratory for Particle Physics and Cosmology, Harvard University, Cambridge, MA USA; 58 Kirchhoff-Institut für Physik, Ruprecht-Karls-Universität Heidelberg, Heidelberg, Germany; Physikalisches Institut, Ruprecht-Karls-Universität Heidelberg, Heidelberg, Germany; ZITI Institut für Technische Informatik, Ruprecht-Karls-Universität Heidelberg, Mannheim, Germany; 59 Faculty of Applied Information Science, Hiroshima Institute of Technology, Hiroshima, Japan; 60 Department of Physics, Indiana University, Bloomington, IN USA; 61 Institut für Astro- und Teilchenphysik, Leopold-Franzens-Universität, Innsbruck, Austria; 62 University of Iowa, Iowa City, IA USA; 63 Department of Physics and Astronomy, Iowa State University, Ames, IA USA; 64 Joint Institute for Nuclear Research, JINR Dubna, Dubna, Russia; 65 KEK, High Energy Accelerator Research Organization, Tsukuba, Japan; 66 Graduate School of Science, Kobe University, Kobe, Japan; 67 Faculty of Science, Kyoto University, Kyoto, Japan; 68 Kyoto University of Education, Kyoto, Japan; 69 Department of Physics, Kyushu University, Fukuoka, Japan; 70 Instituto de Física La Plata, Universidad Nacional de La Plata and CONICET, La Plata, Argentina; 71 Physics Department, Lancaster University, Lancaster, UK; 72 INFN Sezione di Lecce, Lecce, Italy; Dipartimento di Matematica e Fisica, Università del Salento, Lecce, Italy; 73 Oliver Lodge Laboratory, University of Liverpool, Liverpool, UK; 74 Department of Physics, Jožef Stefan Institute and University of Ljubljana, Ljubljana, Slovenia; 75 School of Physics and Astronomy, Queen Mary University of London, London, UK; 76 Department of Physics, Royal Holloway University of London, Surrey, UK; 77 Department of Physics and Astronomy, University College London, London, UK; 78 Louisiana Tech University, Ruston, LA USA; 79 Laboratoire de Physique Nucléaire et de Hautes Energies, UPMC and Université Paris-Diderot and CNRS/IN2P3, Paris, France; 80 Fysiska institutionen, Lunds universitet, Lund, Sweden; 81 Departamento de Fisica Teorica C-15, Universidad Autonoma de Madrid, Madrid, Spain; 82 Institut für Physik, Universität Mainz, Mainz, Germany; 83 School of Physics and Astronomy, University of Manchester, Manchester, UK; 84 CPPM, Aix-Marseille Université and CNRS/IN2P3, Marseille, France; 85 Department of Physics, University of Massachusetts, Amherst, MA USA; 86 Department of Physics, McGill University, Montreal, QC Canada; 87 School of Physics, University of Melbourne, Parkville, VIC Australia; 88 Department of Physics, The University of Michigan, Ann Arbor, MI USA; 89 Department of Physics and Astronomy, Michigan State University, East Lansing, MI USA; 90 INFN Sezione di Milano, Milan, Italy; Dipartimento di Fisica, Università di Milano, Milan, Italy; 91 B.I. Stepanov Institute of Physics, National Academy of Sciences of Belarus, Minsk, Republic of Belarus; 92 National Scientific and Educational Centre for Particle and High Energy Physics, Minsk, Republic of Belarus; 93 Department of Physics, Massachusetts Institute of Technology, Cambridge, MA USA; 94 Group of Particle Physics, University of Montreal, Montreal, QC Canada; 95 P.N. Lebedev Institute of Physics, Academy of Sciences, Moscow, Russia; 96 Institute for Theoretical and Experimental Physics (ITEP), Moscow, Russia; 97 Moscow Engineering and Physics Institute (MEPhI), Moscow, Russia; 98 D.V. Skobeltsyn Institute of Nuclear Physics, M.V. Lomonosov Moscow State University, Moscow, Russia; 99 Fakultät für Physik, Ludwig-Maximilians-Universität München, Munich, Germany; 100 Max-Planck-Institut für Physik (Werner-Heisenberg-Institut), Munich, Germany; 101 Nagasaki Institute of Applied Science, Nagasaki, Japan; 102 Graduate School of Science and Kobayashi-Maskawa Institute, Nagoya University, Nagoya, Japan; 103 INFN Sezione di Napoli, Naples, Italy; Dipartimento di Fisica, Università di Napoli, Naples, Italy; 104 Department of Physics and Astronomy, University of New Mexico, Albuquerque, NM USA; 105 Institute for Mathematics, Astrophysics and Particle Physics, Radboud University Nijmegen/Nikhef, Nijmegen, The Netherlands; 106 Nikhef National Institute for Subatomic Physics and University of Amsterdam, Amsterdam, The Netherlands; 107 Department of Physics, Northern Illinois University, DeKalb, IL USA; 108 Budker Institute of Nuclear Physics, SB RAS, Novosibirsk, Russia; 109 Department of Physics, New York University, New York, NY USA; 110 Ohio State University, Columbus, OH USA; 111 Faculty of Science, Okayama University, Okayama, Japan; 112 Homer L. Dodge Department of Physics and Astronomy, University of Oklahoma, Norman, OK USA; 113 Department of Physics, Oklahoma State University, Stillwater, OK USA; 114 RCPTM, Palacký University, Olomouc, Czech Republic; 115 Center for High Energy Physics, University of Oregon, Eugene, OR USA; 116 LAL, Université Paris-Sud and CNRS/IN2P3, Orsay, France; 117 Graduate School of Science, Osaka University, Osaka, Japan; 118 Department of Physics, University of Oslo, Oslo, Norway; 119 Department of Physics, Oxford University, Oxford, UK; 120 INFN Sezione di Pavia, Pavia, Italy; Dipartimento di Fisica, Università di Pavia, Pavia, Italy; 121 Department of Physics, University of Pennsylvania, Philadelphia, PA USA; 122 Petersburg Nuclear Physics Institute, Gatchina, Russia; 123 INFN Sezione di Pisa, Pisa, Italy; Dipartimento di Fisica E. Fermi, Università di Pisa, Pisa, Italy; 124 Department of Physics and Astronomy, University of Pittsburgh, Pittsburgh, PA USA; 125 Laboratorio de Instrumentacao e Fisica Experimental de Particulas, LIP, Lisbon, Portugal; Faculdade de Ciências, Universidade de Lisboa, Lisbon, Portugal; Department of Physics, University of Coimbra, Coimbra, Portugal; Centro de Física Nuclear da Universidade de Lisboa, Lisbon, Portugal; Departamento de Fisica, Universidade do Minho, Braga, Portugal; Departamento de Fisica Teorica y del Cosmos and CAFPE, Universidad de Granada, Granada, Spain; Dep Fisica and CEFITEC of Faculdade de Ciencias e Tecnologia, Universidade Nova de Lisboa, Caparica, Portugal; 126 Institute of Physics, Academy of Sciences of the Czech Republic, Prague, Czech Republic; 127 Czech Technical University in Prague, Prague, Czech Republic; 128 Faculty of Mathematics and Physics, Charles University in Prague, Prague, Czech Republic; 129 State Research Center Institute for High Energy Physics, Protvino, Russia; 130 Particle Physics Department, Rutherford Appleton Laboratory, Didcot, UK; 131 Physics Department, University of Regina, Regina, SK Canada; 132 Ritsumeikan University, Kusatsu, Shiga Japan; 133 INFN Sezione di Roma, Rome, Italy; Dipartimento di Fisica, Sapienza Università di Roma, Rome, Italy; 134 INFN Sezione di Roma Tor Vergata, Rome, Italy; Dipartimento di Fisica, Università di Roma Tor Vergata, Rome, Italy; 135 INFN Sezione di Roma Tre, Rome, Italy; Dipartimento di Matematica e Fisica, Università Roma Tre, Rome, Italy; 136 Faculté des Sciences Ain Chock, Réseau Universitaire de Physique des Hautes Energies-Université Hassan II, Casablanca, Morocco; Centre National de l’Energie des Sciences Techniques Nucleaires, Rabat, Morocco; LPHEA-Marrakech, Faculté des Sciences Semlalia, Université Cadi Ayyad, Marrakech, Morocco; LPTPM, Faculté des Sciences, Université Mohamed Premier, Oujda, Morocco; Faculté des Sciences, Université Mohammed V-Agdal, Rabat, Morocco; 137 DSM/IRFU (Institut de Recherches sur les Lois Fondamentales de l’Univers), CEA Saclay (Commissariat à l’Energie Atomique et aux Energies Alternatives), Gif-sur-Yvette, France; 138 Santa Cruz Institute for Particle Physics, University of California Santa Cruz, Santa Cruz, CA USA; 139 Department of Physics, University of Washington, Seattle, WA USA; 140 Department of Physics and Astronomy, University of Sheffield, Sheffield, UK; 141 Department of Physics, Shinshu University, Nagano, Japan; 142 Fachbereich Physik, Universität Siegen, Siegen, Germany; 143 Department of Physics, Simon Fraser University, Burnaby, BC Canada; 144 SLAC National Accelerator Laboratory, Stanford, CA USA; 145 Faculty of Mathematics, Physics and Informatics, Comenius University, Bratislava, Slovak Republic; Department of Subnuclear Physics, Institute of Experimental Physics of the Slovak Academy of Sciences, Kosice, Slovak Republic; 146 Department of Physics, University of Cape Town, Cape Town, South Africa; Department of Physics, University of Johannesburg, Johannesburg, South Africa; School of Physics, University of the Witwatersrand, Johannesburg, South Africa; 147 Department of Physics, Stockholm University, Stockholm, Sweden; The Oskar Klein Centre, Stockholm, Sweden; 148 Physics Department, Royal Institute of Technology, Stockholm, Sweden; 149 Departments of Physics and Astronomy and Chemistry, Stony Brook University, Stony Brook, NY USA; 150 Department of Physics and Astronomy, University of Sussex, Brighton, UK; 151 School of Physics, University of Sydney, Sydney, Australia; 152 Institute of Physics, Academia Sinica, Taipei, Taiwan; 153 Department of Physics, Technion, Israel Institute of Technology, Haifa, Israel; 154 Raymond and Beverly Sackler School of Physics and Astronomy, Tel Aviv University, Tel Aviv, Israel; 155 Department of Physics, Aristotle University of Thessaloniki, Thessaloniki, Greece; 156 Department of Physics, International Center for Elementary Particle Physics, The University of Tokyo, Tokyo, Japan; 157 Graduate School of Science and Technology, Tokyo Metropolitan University, Tokyo, Japan; 158 Department of Physics, Tokyo Institute of Technology, Tokyo, Japan; 159 Department of Physics, University of Toronto, Toronto, ON Canada; 160 TRIUMF, Vancouver, BC, Canada; Department of Physics and Astronomy, York University, Toronto, ON Canada; 161 Faculty of Pure and Applied Sciences, University of Tsukuba, Tsukuba, Japan; 162 Department of Physics and Astronomy, Tufts University, Medford, MA USA; 163 Centro de Investigaciones, Universidad Antonio Narino, Bogota, Colombia; 164 Department of Physics and Astronomy, University of California Irvine, Irvine, CA USA; 165 Sezione di Trieste, INFN Gruppo Collegato di Udine, Udine, Italy; ICTP, Trieste, Italy; Dipartimento di Chimica, Fisica e Ambiente, Università di Udine, Udine, Italy; 166 Department of Physics, University of Illinois, Urbana, IL USA; 167 Department of Physics and Astronomy, University of Uppsala, Uppsala, Sweden; 168 Departamento de Física Atómica, Molecular y NuclearDepartamento de Física Atómica, Molecular y Nuclear, Instituto de Física Corpuscular (IFIC), Departamento de Ingeniería Electrónica, Instituto de Microelectrónica de Barcelona (IMB-CNM), University of Valencia and CSIC, Valencia, Spain; 169 Department of Physics, University of British Columbia, Vancouver, BC Canada; 170 Department of Physics and Astronomy, University of Victoria, Victoria, BC Canada; 171 Department of Physics, University of Warwick, Coventry, UK; 172 Waseda University, Tokyo, Japan; 173 Department of Particle Physics, The Weizmann Institute of Science, Rehovot, Israel; 174 Department of Physics, University of Wisconsin, Madison, WI USA; 175 Fakultät für Physik und Astronomie, Julius-Maximilians-Universität, Würzburg, Germany; 176 Fachbereich C Physik, Bergische Universität Wuppertal, Wuppertal, Germany; 177 Department of Physics, Yale University, New Haven, CT USA; 178 Yerevan Physics Institute, Yerevan, Armenia; 179 Centre de Calcul de l’Institut National de Physique Nucléaire et de Physique des Particules (IN2P3), Villeurbanne, France; 180 CERN, 1211 Geneva 23, Switzerland

## Abstract

The performance of the ATLAS muon trigger system is evaluated with proton–proton collision data collected in 2012 at the Large Hadron Collider at a centre-of-mass energy of 8 TeV. It is primarily evaluated using events containing a pair of muons from the decay of $Z$ bosons. The efficiency of the single-muon trigger is measured for muons with transverse momentum $25 < p_\mathrm{{T}}< 100$ GeV, with a statistical uncertainty of less than 0.01 % and a systematic uncertainty of 0.6 %. The $p_\mathrm{{T}}$ range for efficiency determination is extended by using muons from decays of $J/\psi$ mesons, $W$ bosons, and top quarks. The muon trigger shows highly uniform and stable performance. The performance is compared to the prediction of a detailed simulation.

## Introduction

The presence of prompt muons in the final state is a distinctive signature for many physics processes studied in collisions of high energy protons at the LHC. These studies, which led to the discovery of the Higgs boson [1, 2], include measurements of its properties, searches for new phenomena, as well as measurements of Standard Model processes, such as the production of electroweak bosons and top quarks. Therefore, a high-performance muon trigger is essential. In parallel, a good simulation of trigger performance is necessary.

There are many challenges in designing and implementing triggers which select $pp$ interactions with muons in the final state with high efficiency and low transverse momentum, $p_\mathrm{{T}}$, thresholds in the presence of high background conditions. The ATLAS design deploys a three-level, multi-pronged strategy with,

1. custom trigger electronics at Level-1,
2. dedicated fast algorithms to reconstruct muons and estimate their parameters at Level-2,
3. novel techniques to retain high efficiency at the event-filter while utilising offline tracking algorithms.

The Level-2 and event-filter together are called the High Level Trigger. In order to address a wide variety of physics topics, ATLAS has developed a suite of triggers designed to select muons. The single-muon trigger with $p_\mathrm{{T}}$ threshold of 24 GeV is used in many physics analyses. In addition, muon triggers in combination with electrons, jets and missing transverse momentum, as well as moderate-$p_\mathrm{{T}}$ multi-muon triggers, increase sensitivity for various physics topics which benefit from a lower $p_\mathrm{{T}}$ threshold. For the $B$-physics program, various low-$p_\mathrm{{T}}$ multi-muon triggers are used with a special configuration that allows a high efficiency also for non-prompt muons.^1^

The ATLAS experiment collected $pp$ collision data in 2012 at a centre-of-mass energy of 8 TeV with a maximum instantaneous luminosity of $7.7 \times 10^{33}$ $\mathrm cm^{-2}$ $\mathrm s^{-1}$. The number of interactions occurring in the same bunch crossing (called pile-up interactions) was about 25 on average. In this paper, the performance of the ATLAS muon trigger is evaluated, primarily using samples containing muon pairs from $Z$-boson decays. The performance of the low-$p_\mathrm{{T}}$ muon trigger is evaluated with samples containing a pair of muons from the decay of $J/\psi$ mesons. The performance for high-$p_\mathrm{{T}}$ muons is evaluated using events containing top-quarks^2^ or $W$ bosons, where a $W$ boson decays into a muon and neutrino.

## Muon trigger

### ATLAS detector

The ATLAS detector is a multi-purpose particle physics apparatus with a forward–backward symmetric cylindrical geometry and near $4\pi$ coverage in solid angle.^3^ The detector consists of four major sub-systems: the inner detector, electromagnetic calorimeter, hadronic calorimeter and muon spectrometer. A detailed description of the ATLAS detector can be found in Ref. [3]. The inner detector measures tracks up to $|\eta |= 2.5$ in an axial magnetic field of 2 T using three types of sub-detectors: a silicon pixel detector closest to the interaction point, a semiconductor tracker surrounding the pixel detector, and a transition radiation straw tube tracker covering $|\eta |< 2.0$ as the outermost part of the inner detector. The calorimeter system covers the pseudorapidity range $|\eta |< 4.9$ and encloses the inner detector. The high-granularity liquid-argon electromagnetic sampling calorimeter is divided into one barrel ($|\eta |< 1.475$) and two endcap components ($1.375 < |\eta |< 3.2$). The hadronic calorimeter is placed directly outside the electromagnetic calorimeter. A steel/scintillator-tile calorimeter provides hadronic coverage in the range $|\eta |< 1.7$. The endcap and forward regions, spanning $1.5< |\eta |< 4.9$, are instrumented with liquid-argon calorimeters. The calorimeters are then surrounded by the muon spectrometer.

### Muon spectrometer

**Fig. 1:**
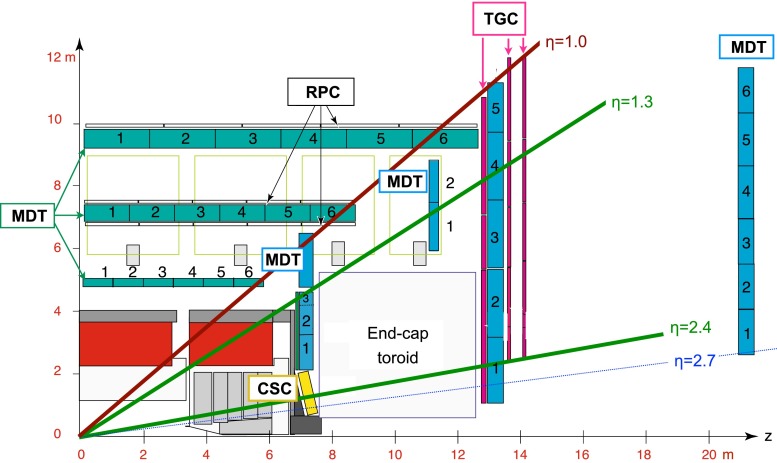
A schematic picture showing a quarter-section of the muon system in a plane containing the beam axis, with monitored drift tube (MDT) and cathode strip (CSC) chambers for momentum determination and resistive plate (RPC) and thin gap (TGC) chambers for triggering

The muon spectrometer is based on three large air-core superconducting toroidal magnet systems (two endcaps and one barrel) providing an average magnetic field of approximately 0.5 T. Figure 1 shows a quarter-section of the muon system in a plane containing the beam axis.

In the central region, the detectors comprise a barrel that is arranged in three concentric cylindrical shells around the beam axis. In the endcap region, muon chambers form large wheels, perpendicular to the $z$-axis. Several detector technologies are utilised to provide both precision tracking and triggering.

The deflection of the muon trajectory in the magnetic field is detected using hits in three layers of precision monitored drift tube (MDT) chambers for $|\eta |< 2$. In the region $2.0 < |\eta |< 2.7$, two layers of MDT chambers in combination with one layer of cathode strip chambers (CSCs) are used. Muons are independently measured in the inner detector and in the muon spectrometer. Three layers of resistive plate chambers (RPCs) in the barrel region ($|\eta |< 1.05$), and three layers of thin gap chambers (TGCs) in the endcap regions ($1.05 < |\eta |< 2.4$) provide the Level-1 muon trigger.

### Level-1 muon trigger

Muons are identified at Level-1 by the spatial and temporal coincidence of hits either in the RPCs or TGCs pointing to the beam interaction region [3, 4]. The Level-1 triggers generated by hits in the RPC require a coincidence of hits in the three layers for the highest three $p_\mathrm{{T}}$ thresholds, and a coincidence of hits in two of the three layers for the rest of thresholds. The Level-1 triggers generated by hits in the TGC require a coincidence of hits in the three layers, except for limited areas in the lowest threshold.

The degree of deviation from the hit pattern expected for a muon with infinite momentum is used to estimate the $p_\mathrm{{T}}$ of the muon with six possible thresholds. The number of muon candidates passing each threshold is used in the conditions for the global Level-1 trigger. Following a global trigger, the $p_\mathrm{{T}}$ thresholds and the corresponding detector regions, region of interest (RoIs), are then sent to the Level-2 and event-filter for further consideration [3, 4]. The typical dimensions of the RoIs are $0.1 \times 0.1$ ($0.03 \times 0.03$) in $\Delta \eta \times \Delta \phi$ in the RPCs (TGCs) [3]. The geometric coverage of the Level-1 trigger is about 99 % in the endcap regions and about 80 % in the barrel region. The limited geometric coverage in the barrel region is due to gaps at around $\eta = 0$ (to provide space for services of the inner detector and calorimeters), the feet and rib support structures of the ATLAS detector and two small elevator shafts in the bottom part of the spectrometer.

### Level-2 muon trigger

The RoI provided by Level-1 enables Level-2 to select the region of the muon detector in which the interesting features reside, therefore reducing the amount of data to be transferred and processed [4]. At Level-2, a track is constructed by adding the data from the MDT chambers to get a more precise estimate of the track parameters, leading to the Level-2 stand-alone-muon [5]. To achieve the needed resolution in sufficiently short time, the $p_\mathrm{{T}}$ of the Level-2 stand-alone-muon is reconstructed with simple parameterised functions. Then, the Level-2 stand-alone-muon is combined with a track found in the inner detector [5]. The closest inner detector track in the $\eta$ and $\phi$ planes is selected as the best matching track. The $p_\mathrm{{T}}$ value is refined by taking the weighted average between that of the Level-2 stand-alone-muon and of the inner detector track, leading to the so called Level-2 combined-muon.

### Event-filter muon trigger

Muons in the event-filter are found by two different procedures. The first focuses on RoIs defined by the Level-1 and Level-2 steps described above and is referred to as the RoI-based method. The second procedure searches the full detector without using the information from the previous levels and is referred to as the full-scan method.

In the RoI-based method, muon candidates are first formed by using the muon detectors (called event-filter stand-alone-muons), and are subsequently combined with inner detector tracks leading to event-filter combined-muons. If no combined-muon is formed, muon candidates are searched for by extrapolating inner detector tracks to the muon detectors. If there are corresponding track segments, combined-muons are formed. Additionally, the degree of isolation for the combined-muon is quantified by summing the $p_\mathrm{{T}}$ of inner detector tracks with $p_\mathrm{{T}}^\mathrm{{trk}} > 1$ GeV found in a cone of $\Delta R = \sqrt{(\Delta \phi )^2 + (\Delta \eta )^2} < \Delta R_\mathrm{{cut}}$, centred around the muon candidate after subtracting the $p_\mathrm{{T}}$ of the muon itself ($\Sigma _{\Delta R < \Delta R_\mathrm{{cut}}} p_\mathrm{{T}}^\mathrm{{trk}}$).

The full-scan procedure is used in the event-filter to find additional muons that are not found by the RoI-based method. In the full-scan, muon candidates are first sought in the whole of the muon detectors, and then inner detector tracks are reconstructed in the whole of the inner detectors. Combined pairs of these inner detector and muon detector tracks form muon candidates called event-filter full-scan-muons.

### Trigger selection criteria

The trigger system is configured to use a large set of selection criteria for each event. Each criterion consists of sequential selections at Level-1, Level-2 and the event-filter, and is referred to as trigger in this paper for simplicity. An event has to satisfy at least one of the triggers in order to be recorded.

Table 1 shows the Level-1 thresholds and the muon triggers discussed in this paper. For all trigger levels, the naming scheme typically follows a convention whereby the number that follows “mu” denotes the transverse momentum threshold and the letters, or combination of letters, characterize the muon type [isolated (i), stand alone (SA), found by full scan (FS)] and/or its origin.

The Level-1 thresholds were optimised to give an efficiency at the designated threshold that is typically 95 % of the maximum efficiency achieved well above the threshold.

**Table 1:** Level-1 $p_\mathrm{{T}}$ thresholds and muon triggers. The sequence shows the requirements at Level-1, in the event-filter or at higher level trigger which then includes Level-2. The requirements at Level-2 are omitted for the single- and multi-muon triggers, as they are looser than those in the event-filter. The applied $p_\mathrm{{T}}$ and isolation requirements are also shown

Level-1	$p_\mathrm{{T}}$ threshold (GeV)	Number of layers in coincidence	
MU4	4	2 (3 in limited areas in the endcap region)	
MU6	6	2 (3 in the endcap region)	
MU10	10	2 (3 in the endcap region)	
MU11	10	3	
MU15	15	3	
MU20	20	3	

Single muon trigger	Level-1	Event-filter	
mu6	MU6	One or more combined-muon with $p_\mathrm{{T}}> 6$ GeV	
mu13	MU10	One or more combined-muon with $p_\mathrm{{T}}> 13$ GeV	
mu18	MU15	One or more combined-muon with $p_\mathrm{{T}}> 18$ GeV	
mu24i	MU15	One or more combined-muon with $p_\mathrm{{T}}> 24$ GeV and $\Sigma _{\Delta R < 0.2} p_\mathrm{{T}}^\mathrm{{trk}} / p_\mathrm{{T}}< 0.12$	
mu36	MU15	One or more combined-muon with $p_\mathrm{{T}}> 36$ GeV	
mu40_SA_barrel	MU15	One or more stand-alone-muon with $p_\mathrm{{T}}> 40$ GeV in $|\eta |<1.05$	

Multi muon trigger	Level-1	Event-filter	
2mu13	Two MU10	Two or more combined-muons with $p_\mathrm{{T}}> 13$ GeV (two or more mu13 triggers)	
mu18_mu8_FS	MU15	One or more combined-muon with $p_\mathrm{{T}}> 18$ GeV (mu18 trigger), and two or more full-scan muons with $p_\mathrm{{T}}> 18$ and $> 8$ GeV	
3mu6	Three MU6	Three or more muons with $p_\mathrm{{T}}> 6$ GeV (three or more mu6 triggers)	

$J/\psi$ tag-and-probe trigger	Level-1	High level trigger	
mu18_$J/\psi$_FS	MU15	(Level-2)	One or more combined-muon with $p_\mathrm{{T}}> 18$ GeV
		(Event-filter)	One or more combined-muon with $p_\mathrm{{T}}> 18$ GeV, and
			at least one pair of combined-muons with a mass consistent with that of $J/\psi$
mu18_$J/\psi$_L2	MU15, MU4	(Level-2)	Two or more combined-muons with $p_\mathrm{{T}}> 18$, $4$ GeV, and
			at least one pair of combined-muons with a mass consistent with that of $J/\psi$
		(Event-filter)	One or more combined-muon with $p_\mathrm{{T}}> 18$ GeV

The triggers described in Table 1 were designed to be as inclusive as possible.

The mu24i trigger is designed to collect isolated muons with $p_\mathrm{{T}}> 25$ GeV with a loose isolation criterion of $\Sigma _{\Delta R < 0.2} p_\mathrm{{T}}^\mathrm{\mathrm{{trk}}} / p_\mathrm{{T}}< 0.12$. The isolation criterion was chosen to retain nearly 100 % efficiency for well isolated muons from the decays of $Z$-bosons while rejecting slightly over half of the muons from heavy flavor, pion and kaon decays.

The mu36 trigger is designed to collect muons with large $p_\mathrm{{T}}$ without making an isolation requirement.

The mu40_SA_barrel trigger is designed to recover possible inefficiency due to muon spectrometer and inner detector combination at large $p_\mathrm{{T}}$, and the decision is based only on muon spectrometer reconstruction. It was active only in the barrel region due to its high rate in the endcaps.

The mu24i, mu36 and mu40_SA_barrel triggers were used without prescale^4^ for the 2012 data taking.

The 2mu13 trigger requires two or more muon candidates, each of which passes the single-muon trigger mu13. The mu18_mu8_FS trigger requires at least one muon candidate which passes the single-muon trigger mu18, and subsequently employs the full-scan algorithm at the event-filter to find two or more muon candidates with $p_\mathrm{{T}}> 18$ and $p_\mathrm{{T}}> 8$ GeV for leading and sub-leading muons, respectively. The full-scan trigger processes the entire detector and utilises more computing resources than the triggers which process only data in one RoI. Computing resources, not bandwidth, is the limiting factor for these triggers. The leading muon was required to have a $p_\mathrm{{T}}$ of at least 18 GeV in the full-scan dimuon triggers for this reason. The 3mu6 trigger requires three or more muon candidates, each of which passes the single-muon trigger mu6. The 2mu13, mu18_mu8_FS and 3mu6 triggers were used without prescale for the 2012 data taking. The dimuon triggers used to select $J/\psi$ decays will be discussed in more detail in Sect. 7.

### Operation in the 2012 data taking

**Fig. 2:**
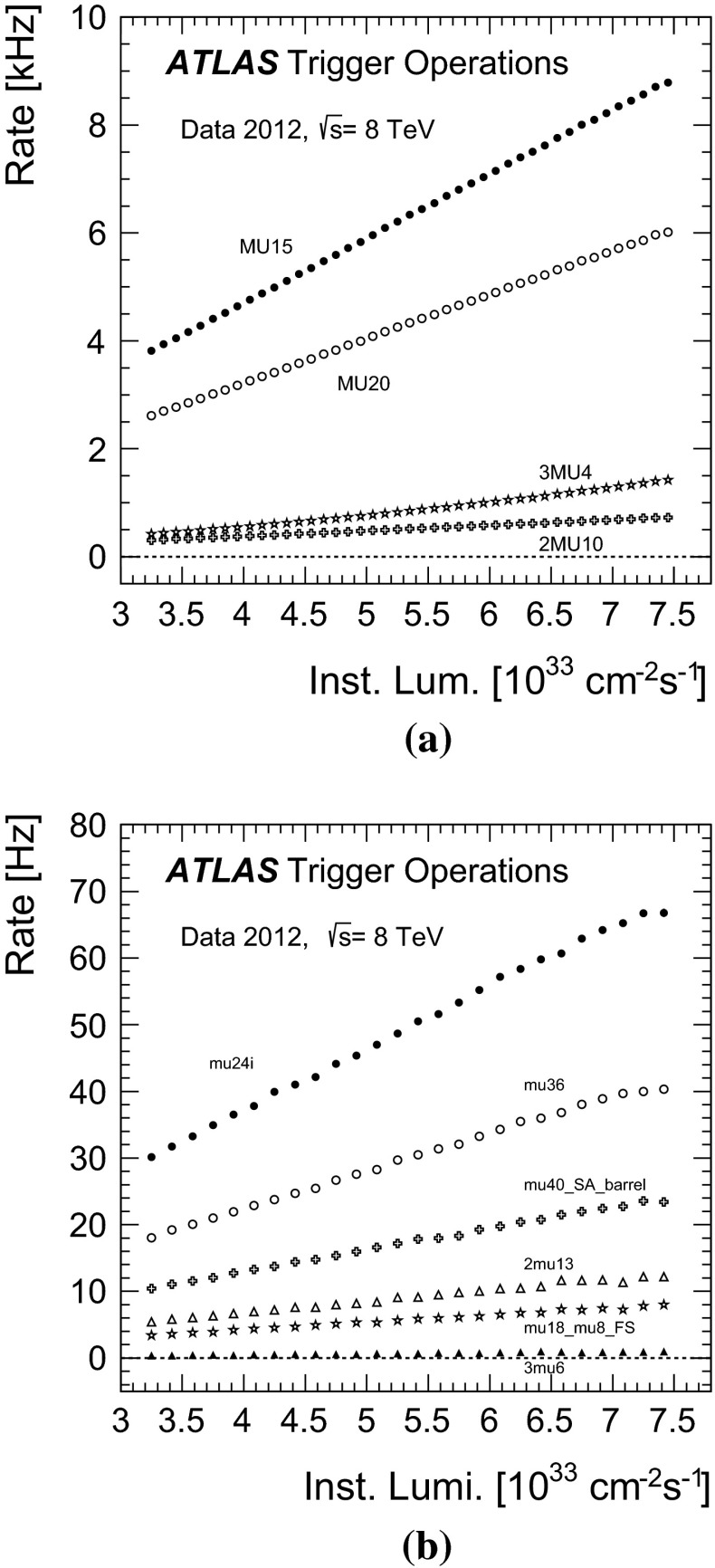
Trigger rates as a function of instantaneous luminosity **a** for selected muon triggers at Level-1 and **b** for selected single- and multi-muon triggers at the event-filter as denoted in the legend (see Table 1 for details)

The typical maximum Level-1 rate was 70 kHz. The event acceptance was reduced at the event-filter which had an output rate of 700 Hz on average (with peaks of about 1 kHz). Of these rates, the single isolated muon trigger mu24i was fired at about 8.5 kHz at Level-1 and at about 65 Hz at the event-filter for an instantaneous luminosity of $7\times 10^{33}$ cm$^{-2}$ s$^{-1}$. Figure 2 shows the rates of the single- and multi-muon triggers as a function of the instantaneous luminosity, separately for the Level-1 and for the event-filter.

They are well described by a linear fit with an approximately zero intercept. This indicates a negligible contribution from effects not related to $pp$ collisions. Typically the trigger rates were reduced by one to two orders of magnitude at Level-2 and by a factor of a few at the event-filter for the single and dimuon triggers. For example the rates were reduced by a factor of 28 at Level-2 (with respect to Level-1) and by a factor of 4.6 at the event-filter (with respect to Level-2) for the mu24i trigger. For the 2mu13 trigger, the rates were reduced by a factor of 71 at Level-2 and by a factor of 1.2 at the event-filter.

During data taking, the performance of the muon trigger was monitored in two stages. For quick online checks during data taking, the coverage in $\eta$–$\phi$ space and the distributions of some kinematic variables were produced by the high level trigger algorithms. A more detailed analysis was performed by calculating efficiencies of triggers during the reconstruction stage of the data processing.

## Data samples and event selection

Several methods are used to measure the muon trigger performance. This section describes the selection requirements used to define the samples needed for the various methods.

### Methods to measure trigger performance

The tag-and-probe method relies on a pair of muons. If one muon has caused the trigger to record the event (called the tag-muon), the other muon serves as a probe (called the probe-muon) to measure the trigger performance without any bias. This method was applied to dimuon decays of $Z$-boson and $J/\psi$ meson candidates. Alternatively, muons contained in events that were recorded by triggers other than the muon trigger can be used as an unbiased sample to evaluate the efficiency of triggering on muons. This method was applied to events with muons from $W$-boson decays, either from top-quark or $W$ $+$ jets production. A trigger based on the missing transverse momentum, as measured with the calorimeter, was used to collect such samples.

Among these four samples, the tag-and-probe method using $Z$ decays provides the most precise determination of the efficiency over a wide range of muon $p_\mathrm{{T}}$ ($10 \lesssim p_\mathrm{{T}}\lesssim 100$ GeV). The tag-and-probe method using $J/\psi$ decays provides a coverage for lower $p_\mathrm{{T}}$ of the muon ($p_\mathrm{{T}}\lesssim 10$ GeV). Muons from $Z$ decays are not frequently found to have $p_\mathrm{{T}}$ $\gtrsim 100$ GeV. Events with muons from top-quark and $W$ $+$ jets production provide supplemental coverage at very high $p_\mathrm{{T}}$ ($p_\mathrm{{T}}\gtrsim 100$ GeV). The muons from top-quark decays tend to have a slightly larger $p_\mathrm{{T}}$ than those from the $Z$ decays due to the larger mass of the top-quark. In the $W$ $+$ jet events, the $W$ may recoil off of one or more high $p_\mathrm{{T}}$ jets. These higher $p_\mathrm{{T}}$ $W$-bosons can then decay into muons with very high $p_\mathrm{{T}}$. In addition, top-quark events and $W$ $+$ jet events offer important cross-checks in the overlapping $p_\mathrm{{T}}$ region that is also covered by the tag-and-probe method using $Z$ decays.

### Data and Monte Carlo samples

Data were considered if recorded under stable beam conditions and with all relevant sub-detector systems fully operational.

The trigger performance observed in the data is compared with the ATLAS Monte Carlo (MC) simulation, which is the same as used for physics analysis. The generated samples were then processed through a simulation of the ATLAS detector based on Geant4 [6, 7]. The environmental backgrounds due to radiation were not simulated. The simulated events are overlaid with additional minimum-bias events generated with Pythia 8 [8] to account for the effect of pile-up interactions.

A sample of $Z$-boson production was generated using Powheg-box [9] interfaced to Pythia 8 [10]. A sample of the production of $J/\psi$ mesons decaying to muon pairs was generated using Pythia 8, requiring at least two muons in the final state having $p_\mathrm{{T}}> 15$ and 2.5 GeV. Similarly to the $Z$-boson production sample, a sample of top and antitop quark pair ($t\bar{t}$) events was generated using Powheg-box interfaced to Pythia 8. Samples of single top-quark events were generated using AcerMC [11] interfaced to Pythia 8 for the $t$-channel production, and using Powheg-box interfaced to Pythia 8 for the $s$- and $Wt$-channel production. Samples of $W$ boson production were generated using Alpgen [12] interfaced to Pythia 8. Samples of dijet events are used for background estimation, and were generated using Pythia 8.

### Offline reconstruction

The offline reconstructed muons are obtained by matching tracks found in the muon spectrometer with those in the inner detector [13]. Muons are required to pass various cuts to ensure a high quality inner detector track and to be in a fiducial region of $|\eta |<2.5$. The muon momentum is calibrated by comparing the dimuon mass of $Z$ boson candidates measured in data and MC [13].

The identification and reconstruction of the electrons, jets, jets containing $B$-hadrons (called $b$-jets), and missing transverse momentum ($E_\mathrm{{T}}^\mathrm{{miss}}$) are necessary for the efficiency measurement with top-quark and $W$-boson candidates.

Electron candidates [14, 15] are required to satisfy $E_\mathrm{{T}}^\mathrm{{el}} > 25$ GeV and $|\eta ^\mathrm{{el}}|<2.47$ excluding $1.37<|\eta ^\mathrm{{el}}|<1.52$, where $E_\mathrm{{T}}^\mathrm{{el}}$ is the transverse energy, and $\eta ^\mathrm{{el}}$ is the pseudorapidity of the electromagnetic cluster of energy deposits in the calorimeter. Candidates are required to be isolated by means of calorimeter- and track-based isolation parameters [16].

Jets are reconstructed using the anti-$k_t$ jet clustering [17] algorithm with a radius parameter $R = 0.4$, running on three-dimensional clusters of cells with significant calorimeter response [18]. Their energies have object-based corrections applied as well as corrections for upstream material, non-instrumented material, and sampling fraction. Jets are required to satisfy $p_\mathrm{{T}}^\mathrm{{jet}}> 25$ GeV and $|\eta ^\mathrm{{jet}}| < 2.5$, where $p_\mathrm{{T}}^\mathrm{{jet}}$ is the transverse momentum, and $\eta ^\mathrm{{jet}}$ is the pseudo-rapidity of the jet. Duplication between electron and jet objects is avoided by removing the jet closest to an electron if their separation is $\Delta R < 0.2$.

The $b$-jets are identified among the reconstructed jets with an artificial neural network using variables that exploit the impact parameter, the secondary vertex and the topology of $b$- and $c$-hadron weak decays [19]. An identification criterion with 70 % efficiency is chosen, as evaluated on jets in a simulated $t\bar{t}$ sample with $p_\mathrm{{T}}> 20 {Ge\,V}$ and $|\eta |< 2.5$.

Hadronically decaying taus are reconstructed using clusters in the electromagnetic and hadronic calorimeter  [3]. A Boosted Decision Tree tau identification method is used to select candiates with a 55–60 % efficiency. Tau candidates are required to have a charge $\pm 1$ and only one or three tracks in a cone of radius $\Delta R < 0.2$.

Photons are identified by electromagnetic cluster of energy deposits in the calorimeter similar to electron identification  [20]. In the case of photons, isolated electromagnetic clusters without matching tracks are classified as unconverted photon candidates. Clusters matched to a pair of tracks that are consistent with the hypothesis of a $\gamma \rightarrow e^+e^-$ conversion process are classified as converted photon candidates.

The $E_\mathrm{{T}}^\mathrm{{miss}}$ is calculated using the reconstructed jets, electrons, muons, $\tau$ leptons, photons, as well as calorimeter energy clusters not associated with these physics objects [21].

In this paper, reconstructed objects (using algorithms applied after the event is recorded) are distinguished from trigger objects (formed either at Level-1, Level-2, or the event-filter during the fast online reconstruction of the event).

### Event selection for the $Z$-boson sample

For the selection of the $Z$-boson sample, events are required to pass either the isolated single-muon trigger mu24i or the single-muon trigger mu36.

A pair of oppositely charged muons with invariant mass, $m_{\mu \mu }$, consistent with the mass of the $Z$ boson, $|m_Z - m_{\mu \mu }| < 10$ GeV, is required. The two muons are required to originate from the same interaction vertex. If one of the two muons has $p_\mathrm{{T}}> 25$ GeV and is isolated, $\Sigma _{\Delta R < 0.2} p_\mathrm{{T}}^\mathrm{{trk}} / p_\mathrm{{T}}< 0.1$, it is a candidate for the tag-muon, and the other muon is a candidate for the corresponding probe-muon. From a pair of muons, two candidate tag- and probe-muons are allowed. Furthermore, the tag-muon candidate must have an angular distance of $\Delta R < 0.1$ to an event-filter combined-muon that passes either the mu24i or mu36 trigger. In addition, the probe-muon candidate has to be isolated, $\Sigma _{\Delta R < 0.2} p_\mathrm{{T}}^\mathrm{{trk}} / p_\mathrm{{T}}< 0.1$.

The probe-muon is matched to a trigger object if it lies within a distance $\Delta R < 0.1$ from an event-filter combined-muon and $\Delta R < 0.5$ from a Level-1 trigger object. The trigger efficiency is defined as the fraction of probe-muons that are associated with at least one trigger muon-object after applying the above criteria.

### Event selection for the $J/\psi$ meson sample

Due to rate restrictions, samples of $J/\psi$ candidates were selected using the two dedicated triggers as in Table 1. One trigger requires a pair of muons found by the event-filter full-scan with a mass consistent with that of the $J/\psi$, with at least one muon with $p_\mathrm{{T}}> 18$ GeV. It is used to determine the efficiency at Level-1 and Level-2. The other trigger requires a pair of muons found by Level-1 and Level-2 with the same requirements as above. It is used to determine the efficiency at the event level with respect to the Level-1 and Level-2. Then the total efficiency can be obtained by multiplying these two partial efficiencies.

All combinations of oppositely charged offline muons are considered as $J/\psi$ candidates if each of the muon tracks satisfies $|d_0| < 0.2$ mm, where $d_0$ is the distance of closest approach between the inner detector track and the proton–proton interaction in the plane transverse to the beam. The two inner detector tracks that are associated with the two muon tracks are refitted under the assumption that they originate from the same vertex. The invariant mass constructed from the refitted tracks, $m_{\mu \mu }$, is required to be consistent with the $J/\psi$ mass, $|m_{J/\psi } - m_{\mu \mu }| < 0.3$ GeV. To enhance the fraction of muons originating from a $J/\psi$ decay a further requirement is made on $L_{xy}$ , the signed two-dimensional decay length of the $J/\psi$. The variable $L_{xy}$ is defined as $L_{xy} \equiv \mathbf {L} \cdot \mathbf {p_\mathrm{{T}}^{J/\psi }} / p_\mathrm{{T}}^{J/\psi }$ with $\mathbf {L}$ being the vector originating from the proton–proton interaction vertex. A requirement of $L_{xy} < 1$ mm is made on the muons. The requirements on $d_0$ and $L_{xy}$ are used to suppress non-prompt muons, such as those from the decays of $B$-hadrons [22].

The fact that these two dedicated triggers were used to select $J/\psi$ candidates implies that the $J/\psi$ mesons are boosted and therefore the spacial distance between the two muons from the decays is small. To ensure correct one-to-one matching between trigger and offline muons, the distance between them is gauged by the separation of the impact points of the tracks at the locations of the RPC and TGC detectors after extrapolation based on the refitted inner detector track parameters. If one of the two muons has $p_\mathrm{{T}}> 18$ GeV and its distance from an event-filter combined-muon that passes the mu18 trigger is within $\Delta R < 0.08$, as evaluated by using the extrapolated positions, it is considered as a tag-muon. If the other muon is beyond the distance of $\Delta R > 0.2$ from the tag-muon, at the extrapolated positions, it is regarded as a probe-muon. The $\Delta R$ cut value is sufficiently large compared to the typical dimensions of the Level-1 trigger segmentation, as described in Sect. 2.3. A probe-muon is matched to trigger objects, if it is within $\Delta R < 0.12$ from a Level-1 muon object and an event-filter combined-muon.

### Selection of top quark and $$W$$$$+$$ jets candidate events

The top quark and $W$ $+$ jets candidate events have to pass a trigger that requires $E_\mathrm{{T}}^\mathrm{{miss}}(\mathrm {calo}) > 80$ GeV, where $E_\mathrm{{T}}^\mathrm{{miss}}(\mathrm {calo})$ is the magnitude of the missing transverse momentum as measured using only the calorimeter information. Several additional cuts are then imposed to remove events with noise bursts in the calorimeters and those with cosmic-ray showers.

The muon candidate is required to have $p_\mathrm{{T}}> 40$ GeV and $|z_0| < 2$ mm, where $z_0$ is the track impact parameter in the $z$-direction with respect to the proton–proton interaction vertex. The probe-muon is required to be isolated from neighbouring jets and energy depositions in the calorimeter. Probe-muons are required to satisfy $\Sigma _{\Delta R < 0.3} p^\mathrm{trk}_T/p_\mathrm{{T}}< 0.05$ and $\Delta R_{\min } (\mathrm {jet,muon}) > 0.4$, where $\Delta R_{\min } (\mathrm {jet,muon})$ is the minimum distance between the muon and any jet. In addition, no other muon with $p_\mathrm{{T}}> 25$ GeV is allowed.

Events are further required to have $E_\mathrm{{T}}^\mathrm{{miss}}> 20$ GeV and $m_\mathrm{{T}}^{W}$+$E_\mathrm{{T}}^\mathrm{{miss}}> 60$ GeV, where $m_\mathrm{{T}}^{W}$ is the transverse mass^5^ of the $W$ candidate. The $W$ is reconstructed with four-vectors of the $E_\mathrm{{T}}^\mathrm{{miss}}$ and the muon.

For the top quark sample, there must be at least three jets with at least one $b$-jet. For the $W$ sample, there must be one or two jets with no $b$-jets. Events with an electron are rejected.

## Trigger purity

The trigger purity is defined as the fraction of muon triggers that can be associated to an offline muon. The $\Delta R$ distance between the trigger object and the offline muon was used to define this matching.

**Fig. 3:**
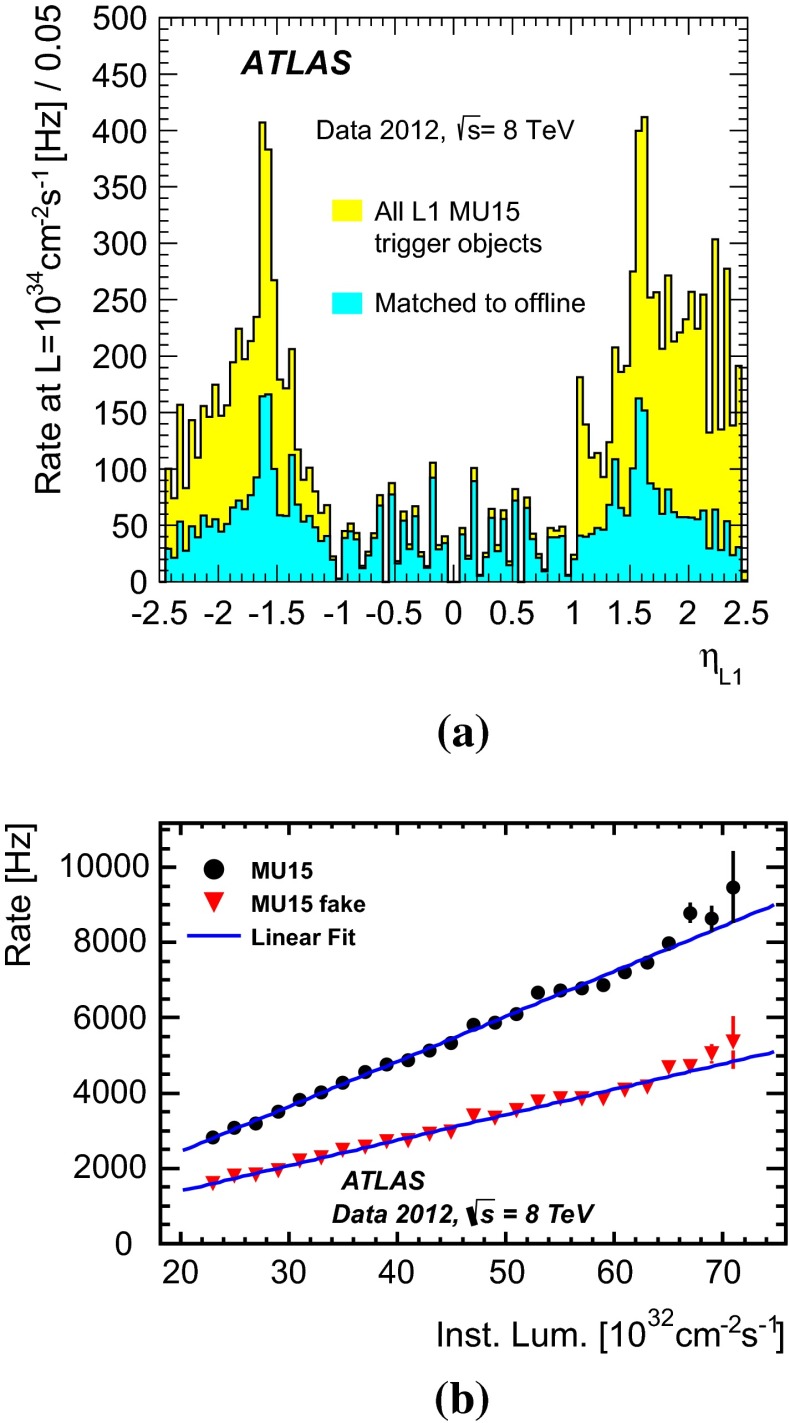
Trigger rate of the Level-1 MU15 as a function of **a** pseudorapidity $\eta _\mathrm{{L_1}}$ of all the trigger objects (*light histogram*) and of the ones associated with offline reconstructed muons (*dark histogram*) and **b** instantaneous luminosity, for all triggers (*dots*) and for the fake ones not-associated with offline-reconstructed muons (*triangles*) with the *lines* representing the results of the corresponding linear fits

The $\eta$ distribution of the Level-1 MU15 object that seeds the mu24i event-filter is shown in Fig. 3a for all triggers and for those associated with a reconstructed offline muon. No explicit cut on offline muon $p_\mathrm{{T}}$ was applied in the association between trigger and offline objects. Figure 3 shows that the Level-1 rate is dominated by triggers without associated offline muons (called fake triggers). The overall trigger purity (fraction of Level-1 rate from true muons ) is 40 %. Most of the Level-1 fakes originates in the end-cap. The cause of these fakes in the endcap region was extensively investigated [23], and is understood as mainly due to charged particles, for instance protons, produced in large amounts of dense material such as the toroid coils and shields. Figure 3b shows the MU15 trigger rate as a function of the instantaneous luminosity. Also shown is the rate due to fake triggers. The error bars show statistical uncertainties only. Both the total rate and the fake rate at Level-1 scale linearly with the instantaneous luminosity.

**Fig. 4:**
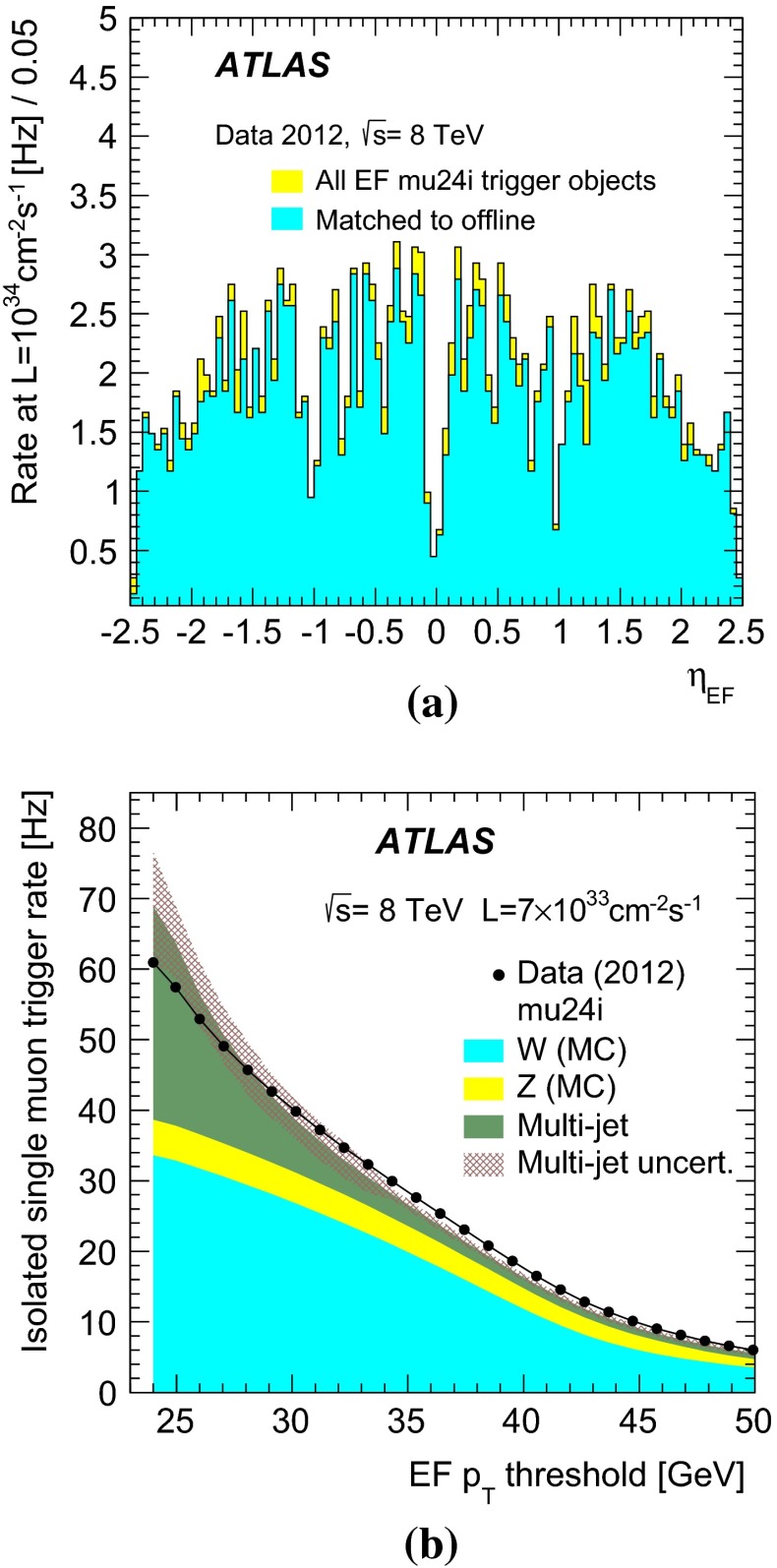
Rate of the isolated single-muon trigger, mu24i, at the event-filter **a** as a function of pseudorapidity $\eta _\mathrm{{EF}}$ for all combined-muons (*light histogram*) and for the ones associated with offline reconstructed muons (*dark histogram*), **b** as a function of the transverse momentum $p_\mathrm{{T}}$ threshold at the event-filter (EF) at an instantaneous luminosity of $7\times 10^{33}$ cm$^{-2}$ s$^{-1}$, for combined-muons in the data (*dots*) compared to the expectations from $W$- and $Z$-bosons production and from the data-driven estimate for multi-jet production, as described in the legend

Figure 4a shows the $\eta$ distribution of the trigger objects recorded with the isolated single-muon trigger at the event-filter. The fake triggers, not associated to an offline reconstructed muon, are rejected by the subsequent High-Level-Trigger decisions, and a purity of about 90 % is achieved. The physics origin of muons at the event-filter is illustrated in Fig. 4b, which shows the expected composition of the trigger rate of the isolated single-muon as a function of the lower threshold value on the muon $p_\mathrm{{T}}$. The vertical scale gives the trigger rate as a function of $p_\mathrm{{T}}$ at an instantaneous luminosity of $7 \times 10^{33}$ cm$^{-2}$ s$^{-1}$. The expectations for $W$ and $Z$ production were evaluated by using MC simulations with their predicted cross sections. Multi-jet production, where one or more jets produce a muon from the decay of a heavy quark or from a pion or kaon decay in flight, also contribute to this rate. The multi-jet contribution was evaluated in a data-driven approach as described below.

A multi-jet enriched control-region is obtained by using events that are triggered by a single-muon trigger with the same $p_\mathrm{{T}}$ threshold but without isolation requirement.^6^ The control-region is defined by inverting the trigger isolation criteria, by requiring at least one jet in an event, and by requiring matching to an offline muon to remove the fake contribution. The multi-jet contribution in the signal region is estimated by the following procedure. The fraction of multi-jet events in the signal region is taken from dijet MC simulation. The total normalization for the multi-jet contribution is then evaluated in the control-region. The contribution to the signal region is then taken as the total estimated multi-jet contribution weighed by the signal fraction from simulation. The uncertainty of this estimation is dominated by the statistical uncertainty in the control-region/signal-region transfer factors from MC simulation, and is shown in Fig. 4b. The rate was evaluated as a function of the $p_\mathrm{{T}}$ threshold on the event-filter combined-muon. As shown in Fig. 4b, at $p_\mathrm{{T}}= 24$ GeV about 60 % of the events triggered by mu24i are due to muons from $W$ and $Z$ production.

## Resolution

The tag-and-probe method applied to $Z$-boson candidates was used to evaluate the quality of the $p_\mathrm{{T}}$, $\eta$ and $\phi$ determination at the event-filter, compared to the offline reconstruction. The online algorithms are nearly identical to the offline versions but have some simplifications in the pattern recognition because of timing constraints. Additionally, the offline reconstruction uses updated calibration and alignment corrections not available at the time the data was recorded. Therefore, finite difference can be expected even when the event-filter combined muon is compared with the offline muon that is also reconstructed by combining the inner detector and muon detectors.

**Fig. 5:**
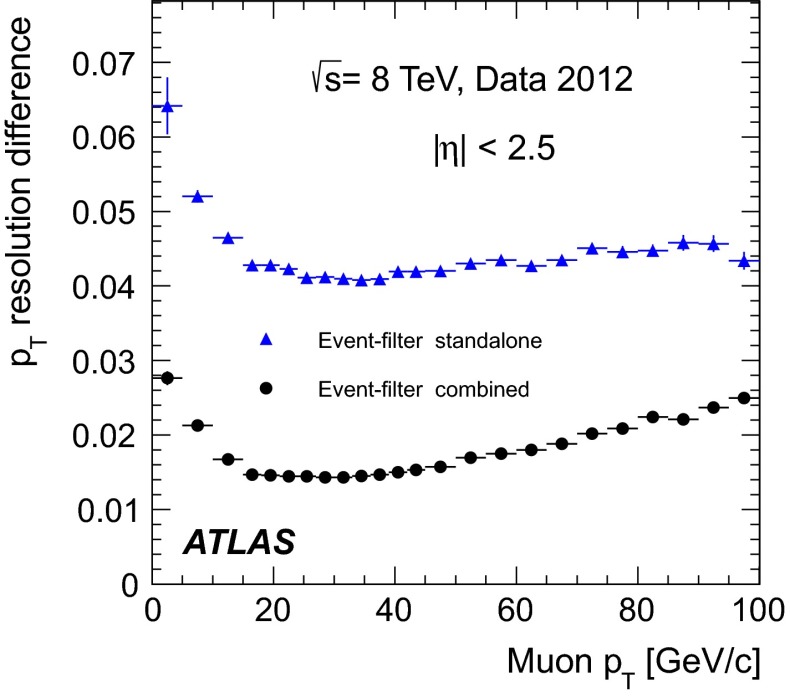
Resolution difference in transverse momentum $p_\mathrm{{T}}$ determination in the offline and in the event-filter reconstruction, as a function of $p_\mathrm{{T}}$ of the offline muon

The offline momentum resolution is $< 3.5$ % up to transverse momenta $p_{T}$ of 200 GeV and $<$ 10 % up to 1 TeV  [24]. The residual of the trigger-reconstructed $p_\mathrm{{T}}$ with respect to the offline value is defined as $\delta _{p_\mathrm{{T}}} = \frac{1/p^\mathrm{{trigger}}_T - 1/p_\mathrm{{T}}}{1/p_\mathrm{{T}}}$, where $p^\mathrm{{trigger}}_{T}$ is the transverse momentum reconstructed by the trigger, and the $p_\mathrm{{T}}$ is that of the offline muon. The resolution difference between the trigger and offline reconstruction was defined as the standard deviation of a Gaussian function fitted to the $\delta _{p_\mathrm{{T}}}$ distribution. Figure 5 shows the $p_\mathrm{{T}}$ resolution differences, as a function of the offline muon $p_\mathrm{{T}}$, of the event-filter stand-alone and event-filter combined muons in the barrel and endcap regions. The $p_\mathrm{{T}}$ resolution difference is about 2 and 5 % for event-filter combined and event-filter stand-alone muon, respectively.

**Fig. 6:**
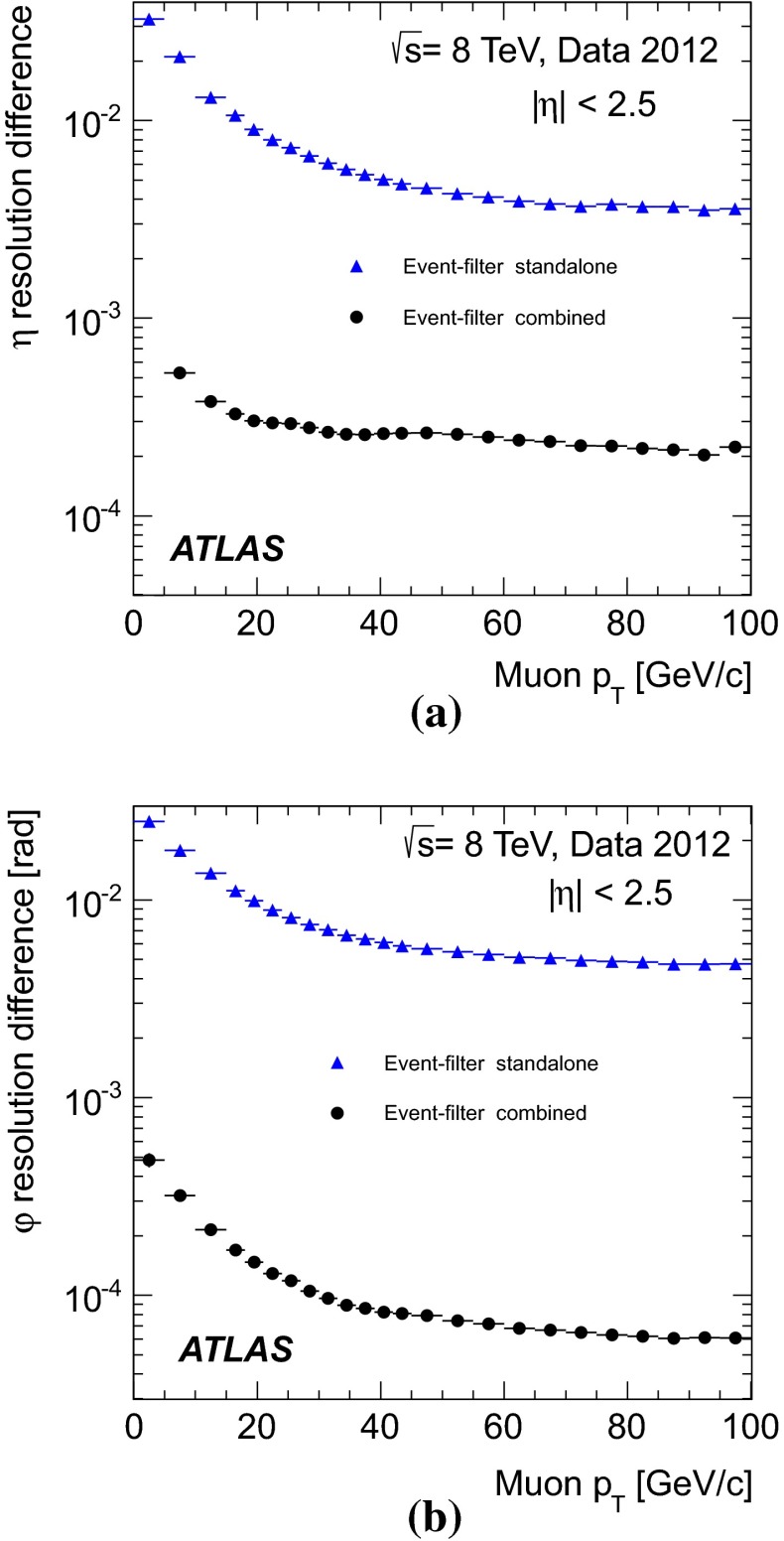
Resolution difference in the **a** pseudorapidity $\eta$ and **b**  azimuthal angle $\phi$ determination in the offline and in the event-filter reconstruction, as a function of $p_\mathrm{{T}}$ of the offline muon

The resolution differences of the $\eta$ and $\phi$ determination were examined similarly by defining the residual as the absolute value of the difference between the trigger and offline reconstructed values. Figure 6 shows the $\eta$ and $\phi$ resolution differences of the event-filter muons. It shows that the trigger–offline matching criterion used in the efficiency measurements, for instance $\Delta R < 0.1$ for the tag-and-probe method using $Z$ bosons (see Sect. 3.4), is sufficiently loose.

## Efficiency measurements with $Z$ boson candidates

In the next several sections, measurements of the efficiency of the muon trigger in different kinematic regions are presented, preceded by a discussion of systematic uncertainties. The efficiency is primarily measured as a function of muon $p_\mathrm{{T}}$. In addition, the efficiency is measured in two-dimensions, for instance in $\eta$ and $\phi$ bins, and compared to the simulated one. To more accurately model data, all ATLAS physics analysis which use events selected with the muon trigger are provided with the ratios of measured to simulated efficiencies to make small corrections to the simulated samples.

### Systematic uncertainty

In the following, sources of systematic uncertainty are discussed and the quoted uncertainty values are presented for the efficiency measured in the region of $25 < p_\mathrm{{T}}< 100$ GeV.

**Fig. 7:**
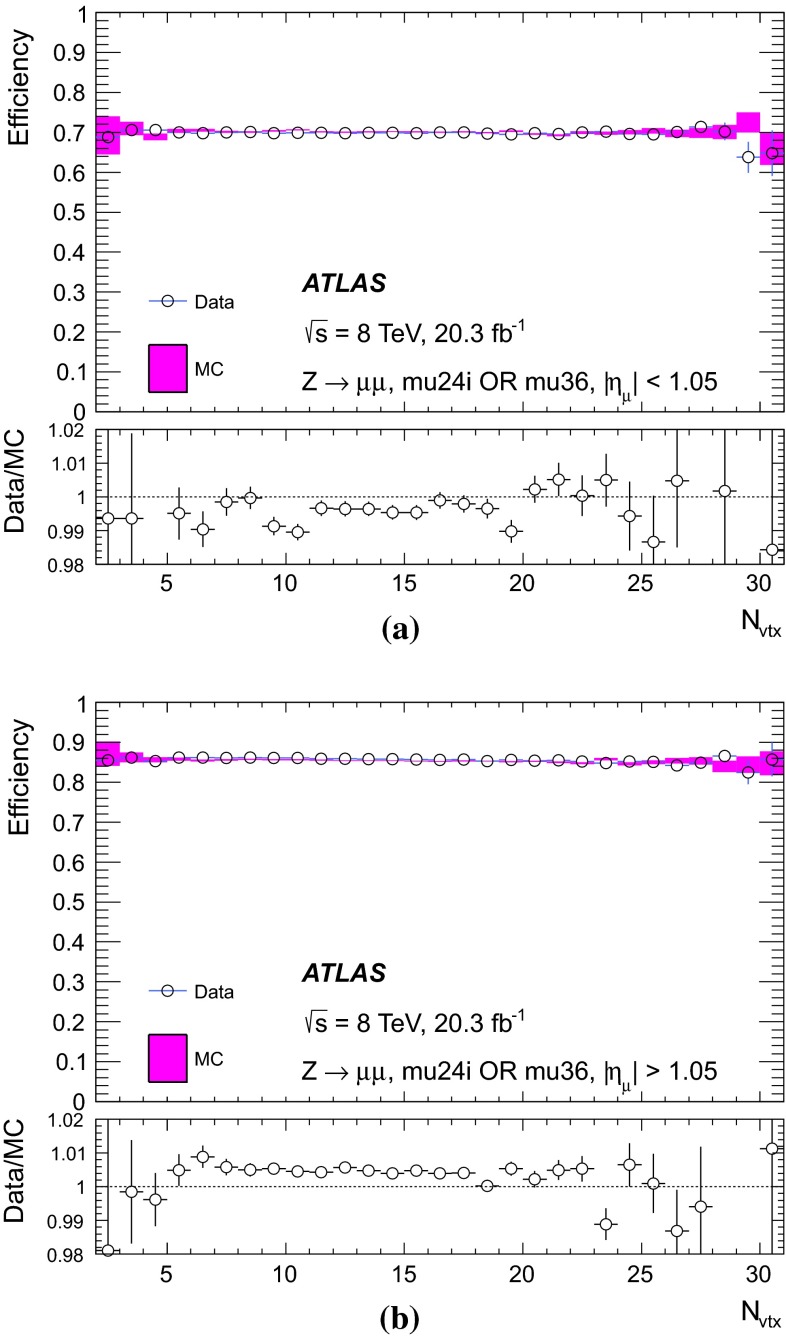
Efficiency to pass either mu24i or mu36 triggers, as a function of the number of reconstructed vertices in an event, N$_\mathrm{{vtx}}$ in **a** the barrel region, and in **b** the endcap region, for data (*dots*) and MC simulation (*bands*). The *lower panels* show the ratio of the efficiencies in data and in MC simulation. The *error bars* reflect statistical uncertainties only

- Dependence on pile-up interactions: the efficiency was measured as a function of the number of reconstructed vertices, Nvtx, separately for data and MC simulation, as shown in Fig. 7. The efficiency is largely independent of the number of pile-up interactions. Separate linear fits to the data and MC simulation were performed in the range from Nvtx $=$ 5 to Nvtx $=$ 30 and extrapolated out to Nvtx $=$ 50. The dependence on the fit range was observed to be negligible. The largest difference observed between the fits in data and MC simulation were observed to be 0.1 (0.5) % in the barrel (endcap). This difference is taken as an estimate of the systematic uncertainty due to the presence of pile-up interactions.
- Correlation between tag- and probe-muons from $Z$ decays: for medium $p_\mathrm{{T}}$, tag- and probe-muons tend to be back-to-back in $\phi$. Since the barrel and endcap have 16-fold and 12-fold symmetries, respectively, this can potentially lead to some bias; a tag-muon from a $Z$ -boson decay inside a highly efficient region of the detector tends to be accompanied by a probe-muon in a region of high efficiency. This effect is evaluated by adding a requirement to the tag and probe pairs to prevent them from being back-to-back, $\Delta \phi \mathrm{{(tag, probe)}} < \pi -$ 0.1, where $\Delta \phi \mathrm{{(tag, probe)}}$ denotes the azimuthal angle between the tag- and probe-muons. The resulting uncertainty in the efficiency determination is 0.3 % (0.2 %) in the barrel (endcap) region.
- Matching between probe-muon and trigger muon: this effect was estimated by changing the $\Delta R$ thresholds of the matching criteria. The change in the efficiency determination was found to be negligible.
- Probe-muon momentum scale and resolution: this effect was estimated by changing the momentum scale and momentum resolution for the probe-muon by their respective uncertainties, as determined from the calibration using $Z$-bosons. The resulting change in efficiency was negligible.
- Probe-muon selection criteria: this effect was estimated by changing, typically by 10 %, the cuts in various selection criteria, leading to negligible changes in the efficiency determination.
- Background contribution: the amount of background was estimated by using the dijet, $t\bar{t}$, and $W$ MC simulations and the effect on the efficiency determination was found to be negligible [25]. Also, varying the $Z$ mass window cut gave negligible effect.
- MC modelling: the sensitivity of the efficiency determination to the MC modelling was tested by comparing samples generated with a different MC generator, namely by adding Sherpa [26]. Again, the change in efficiency was found to be negligible [25].
- Dependence on $p_\mathrm{{T}}$: after correcting the MC efficiency in $\eta$ and $\phi$ so as to reproduce the one observed in the data , any residual deviations between data and MC in the $p_\mathrm{{T}}$ dependence are taken as systematic uncertainty. This resulted in a 0.4 % effect.
- Probe-muon charge dependence: it was estimated by comparing the efficiencies measured with positively charged and negatively charged probe-muons. The estimated uncertainty is 0.2 % in the endcap region.

The individual systematic uncertainties are added in quadrature to obtain the total systematic uncertainty, resulting in 0.6 % for the efficiency measured in the region of $25 < p_\mathrm{{T}}< 100$ GeV.

### Single-muon triggers: mu24i, mu36

**Fig. 8:**
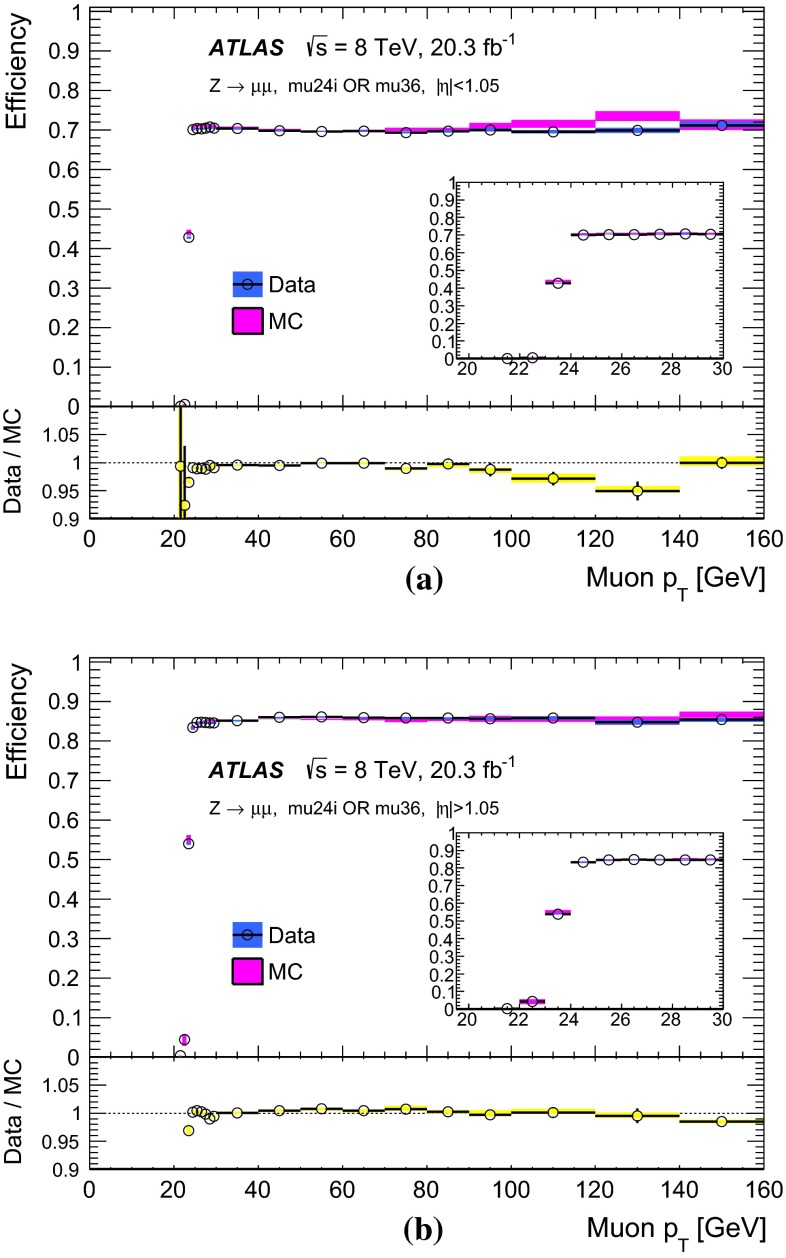
Efficiency of passing either the mu24i or mu36 trigger as a function of the probe-muon transverse momentum $p_\mathrm{{T}}$, for **a** the barrel region and **b** the endcap region, for data (*dots*) and MC simulation (*bands*). The *lower panels* show the ratio of the data and MC efficiencies. The *error bars* include both statistical and systematic uncertainties

Requiring events to pass either the mu24i or the mu36 trigger serves as a general-purpose single-muon triggers for many physics analyses. Figure 8 shows the efficiency to pass either the mu24i or the mu36 trigger as determined in the barrel and endcap regions. The efficiency was measured as a function of the $p_\mathrm{{T}}$ of the reconstructed probe-muon for both data and simulation. The efficiency in the simulation is seen to match that of the data over a wide $p_\mathrm{{T}}$ range. The slight excess in simulation in the $p_\mathrm{{T}}$ bin centred at 130 GeV was studied in detail. High $p_\mathrm{{T}}$ muons from $Z$-boson decays tend to be slightly more forward where there is the largest difference in trigger efficiency between data and simulation.

The efficiency curve turns on sharply around the threshold, reaching a plateau already around $p_\mathrm{{T}}\sim 25$ GeV. In order to quantitatively evaluate the turn-on behaviour and the agreement between data and MC simulation, a fit was made using a Fermi function $f(p_{T})$.^7^ From the fit, the low edge of the efficiency plateau region was defined as the value of $p_\mathrm{{T}}$ for which the efficiency decreases by 1 % from the plateau value. Table 2 shows these evaluated plateau values as well as the location of the low edges of the plateaus. The single-muon trigger that requires either the mu24i or mu36 trigger exhibits a plateau efficiency for physics analysis with muon $p_\mathrm{{T}}> 25$ GeV. The efficiency plateau is smooth at $p_\mathrm{{T}}=36$ GeV indicating that there is no inefficiency due to the isolation requirement in this sample.

**Table 2:** Result of fitting a Fermi function to the efficiency turn-on curve as a function of transverse momentum $p_\mathrm{{T}}$ for the single-muon trigger, for data and MC simulation. The location in $p_\mathrm{{T}}$ of the low edge of the plateau region is defined such that the efficiency decreases by 1 % from the plateau value

	Data		MC	
Trigger	Plateau value (%)	Low edge (GeV)	Plateau value (%)	Low edge (GeV)
Either mu24i or mu36				
Barrel	70.1	24.3	70.3	24.0
Endcap	85.6	24.8	85.3	24.7

**Fig. 9:**
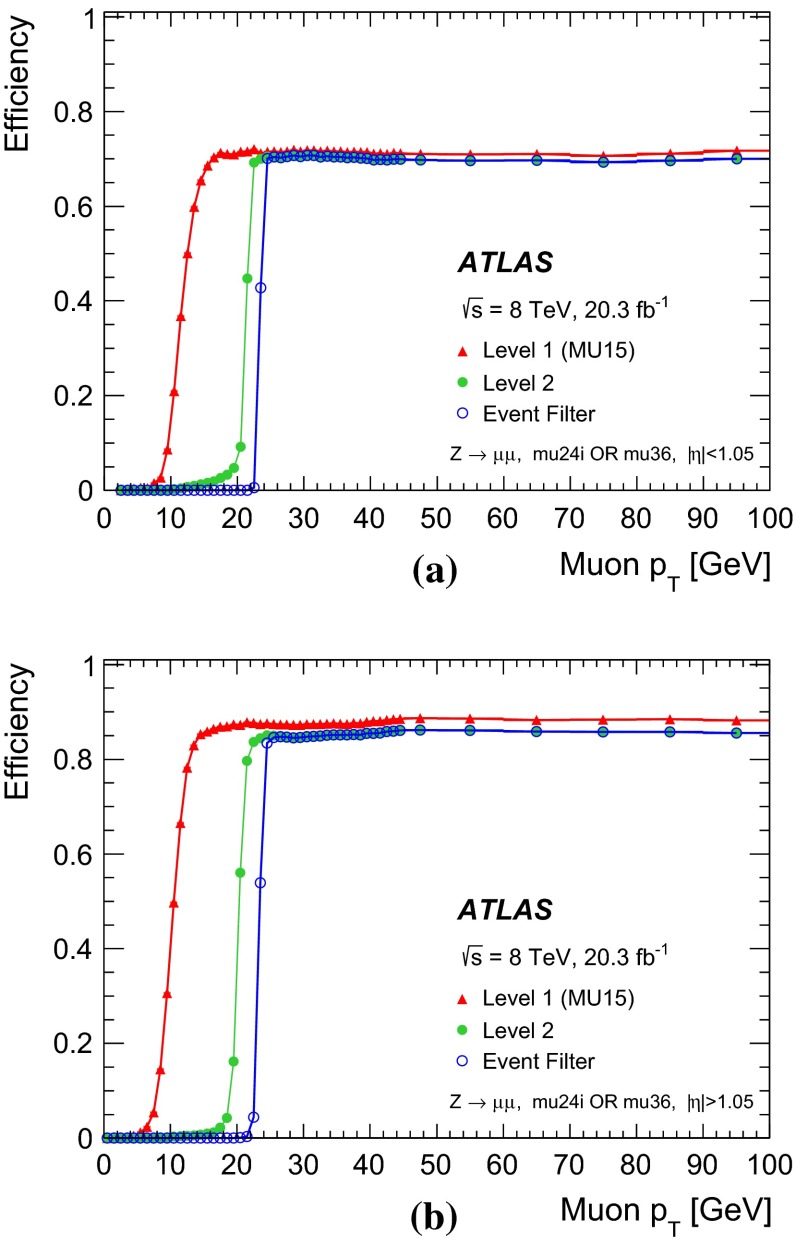
Efficiency of passing either the mu24i or mu36 trigger as functions of the probe-muon transverse momentum $p_\mathrm{{T}}$, for the three trigger levels, Level-1, Level-2 and event-filter, in the data for **a** the barrel region and **b** the endcap region. The *error bars* show the statistical uncertainties only

Figure 9 shows the efficiency of requiring to pass either mu24i or mu36 triggers, as measured separately for the three trigger levels, Level-1, Level-2 and event-filter. The trigger selection becomes tighter and the efficiency turn-on becomes sharper as the trigger level increases. The plateau efficiency is mostly determined by Level-1. The high level trigger efficiency with respect to Level-1 is about 98–99 %.

**Fig. 10:**
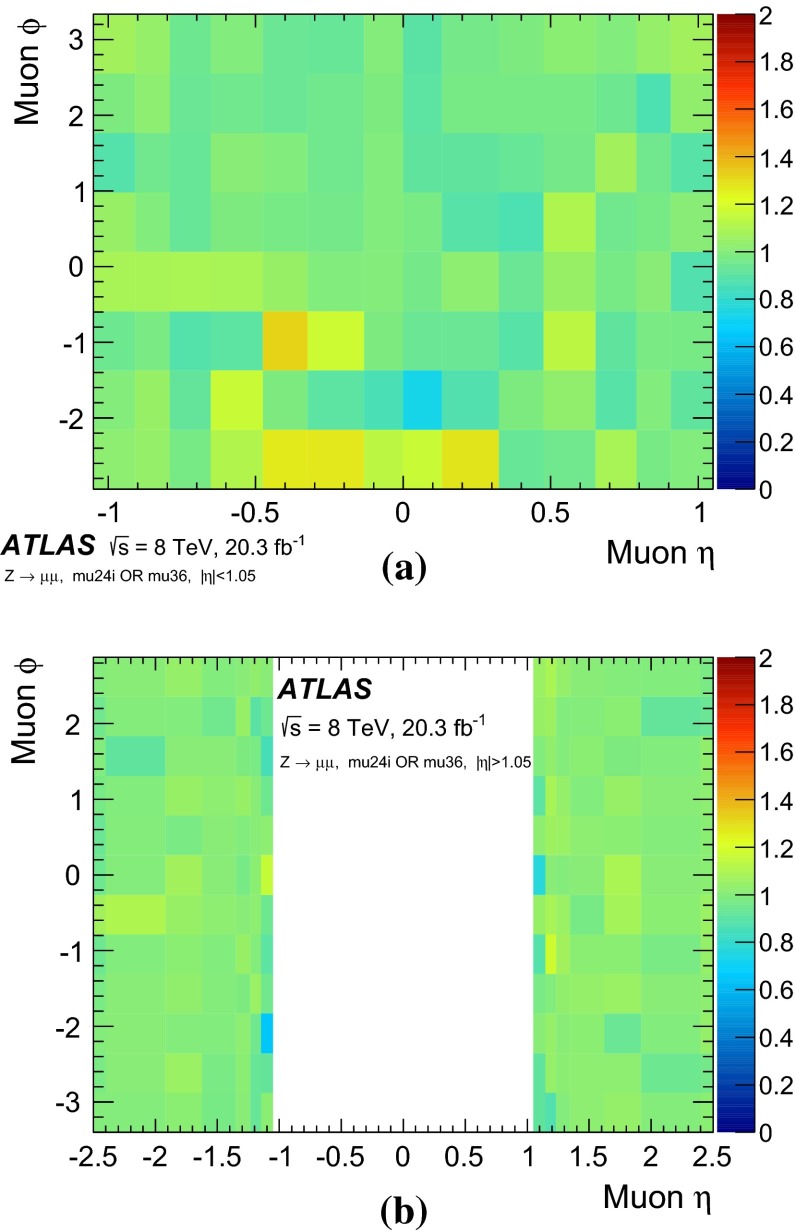
Ratio of the data and MC efficiencies to pass either the mu24i or the mu36 trigger, in bins of the probe-muon $\eta$ and $\phi$ in **a** the barrel region and **b** the endcap region

Figure 10 shows the ratio of the data and MC efficiencies to pass either the mu24i or the mu36 trigger, as determined in bins of $\eta$ and $\phi$ of the probe-muon, for the barrel and endcap regions. The measurement was performed for muons with $p_\mathrm{{T}}> 25$ GeV. The bins in $\eta$ and $\phi$ are fine enough to reflect the hardware segmentation of the Level-1 detectors but coarse enough to have sufficient statistics in each bin. The typical size of the statistical uncertainty is less than 1 %, except for a few specific areas where the uncertainty is about 3 %.

### Other single-muon triggers

**Fig. 11:**
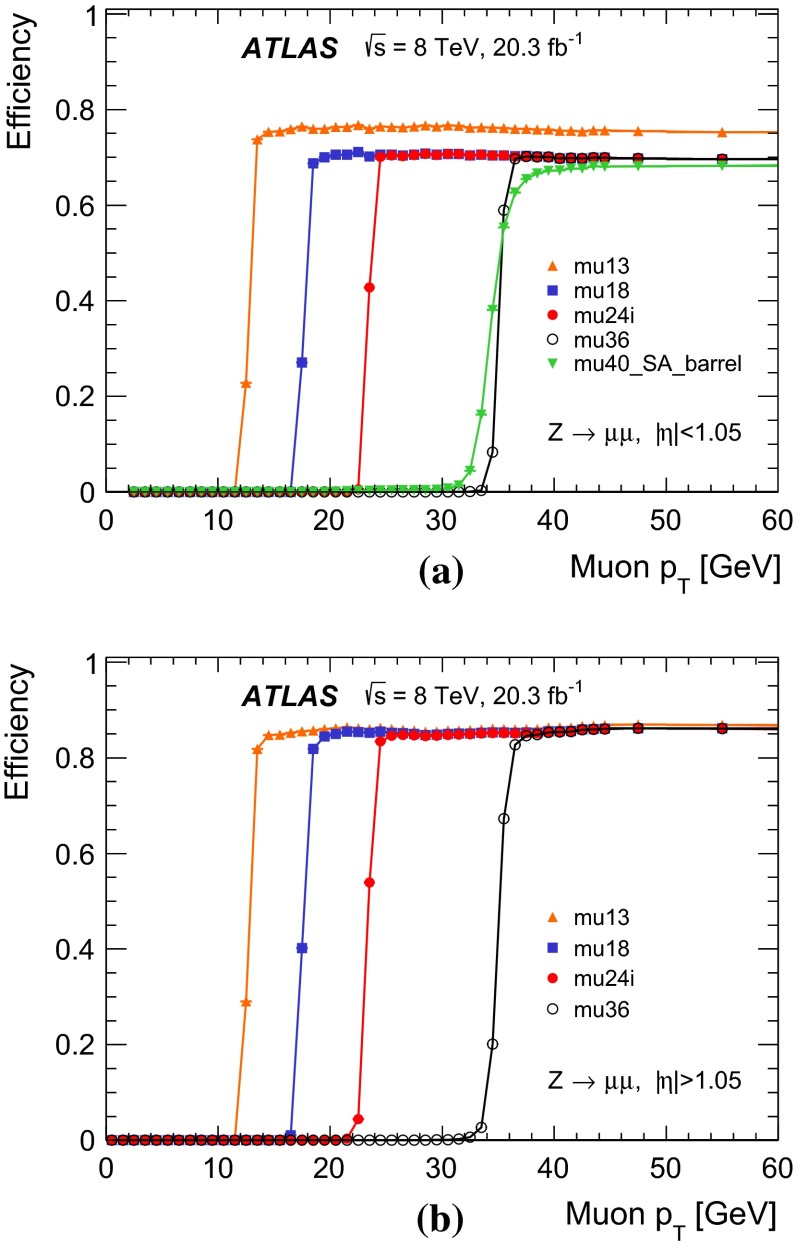
Efficiency of single-muon triggers, mu13, mu18, mu24i, mu36 and mu40_SA_barrel, measured in data as a function of the probe-muon transverse momentum $p_\mathrm{{T}}$, for **a** the barrel region and **b** the endcap region. The *error bars* indicate statistical uncertainties only

Figure 11 shows the efficiencies of the mu36 trigger and of the mu40_SA_barrel trigger, together with that of mu24i trigger, as measured in data. The turn-on behaviour of mu24i and mu36 are sharp, while it is slower at threshold for mu40_SA_barrel. The latter relies only on the information from the muon detectors, and thus the $p_\mathrm{{T}}$ resolution is coarser (see Sect. 5). On the other hand, the requirement to pass either mu36 or mu40_SA_barrel results in about 2 % higher efficiency in the barrel region than achieved when requiring mu36 only, because mu40_SA_barrel does not require an inner detector track match. Therefore, requiring that either the mu36 or mu40_SA_barrel triggers are passed serves as a primary single-muon trigger for any processes that include muons with $p_\mathrm{{T}}\gtrsim 50$ GeV.

Figure 11 also shows the efficiencies of the medium-$p_\mathrm{{T}}$, single-muon triggers, mu13 and mu18. The plateau efficiency of mu13 is about 6 % higher in the barrel region than that of mu18 and other higher-$p_\mathrm{{T}}$ triggers like mu24i. This is because mu13 is seeded from Level-1 MU10, which requires a two-station coincidence, while mu18 and the others are seeded from Level-1 MU15 which requires a three-station coincidence (see Sect. 2.3).

A fit using a Fermi function was performed to quantify the turn-on behaviour of these medium-$p_\mathrm{{T}}$ single-muon triggers. Table 3 shows the evaluated plateau and low edge $p_\mathrm{{T}}$ values for mu13 and mu18. It is seen that the offline cut of muon $p_\mathrm{{T}}> 15 (20)$ GeV is sufficient to ensure the mu13 (mu18) trigger efficiency is described by the plateau value. These middle-$p_\mathrm{{T}}$ triggers are used in various triggers, such as dimuon triggers 2mu13 and mu18_mu8_FS. The efficiencies of the single-muon triggers, mu13 and mu18, are necessary ingredients to calculate the dimuon trigger efficiencies.

**Table 3:** Result of Fermi function fit to the efficiency turn-on curve for the middle-$p_\mathrm{{T}}$ single-muon triggers. The location in $p_\mathrm{{T}}$ of the low edge of the plateau region is defined such that the efficiency decreases by 1 % from the plateau value

	Data		MC	
Trigger	Plateau value (%)	Low edge (GeV)	Plateau value (%)	Low edge (GeV)
mu13				
Barrel	75.8	13.7	75.0	12.8
Endcap	86.4	13.6	86.1	13.4
mu18				
Barrel	70.1	18.2	70.4	18.1
Endcap	85.7	18.7	85.4	18.4

### Full-scan-muon trigger

As described in Sect. 2.6, the mu18_mu8_FS trigger is split into the RoI-based single-muon trigger, mu18, and the full-scan triggers of mu18_FS and mu8_FS. The full-scan trigger efficiencies were evaluated using the same method and sources of systematic uncertainties as for the single-muon trigger (see Sect. 6.1). Only two sources of systematic uncertainties resulted in visible changes in the efficiency, while all others lead to negligible changes.

**Fig. 12:**
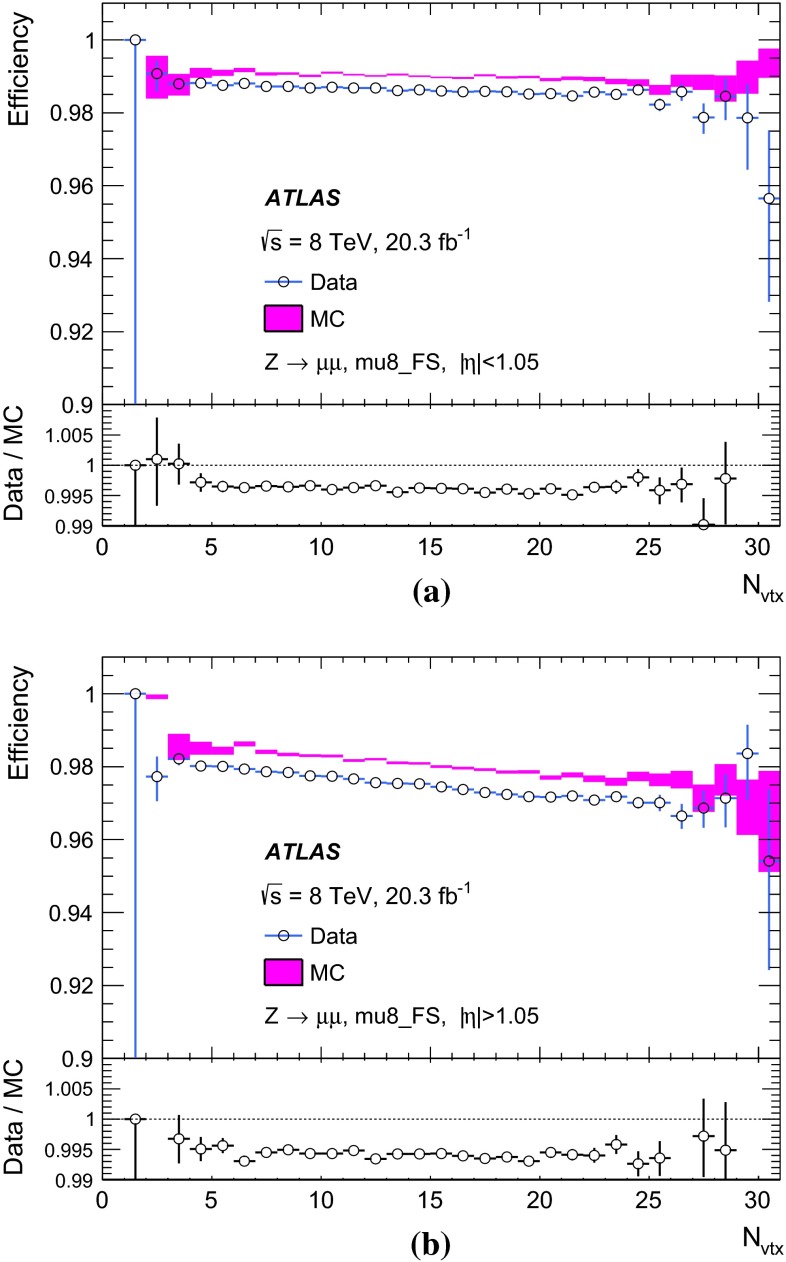
Efficiency of the mu8_FS trigger measured as a function of the reconstructed number of vertices in an event, N$_\mathrm{{vtx}}$ in **a** the barrel region and **b** the endcap region, in the data (*dots*) and in the MC simulation (*bands*) The *lower panels* show the ratio of efficiencies of data and MC simulation. The *error bars* represent statistical uncertainties only

- Dependence on $p_\mathrm{{T}}$: the uncertainty was estimated by comparing data and MC efficiencies as a function of $p_\mathrm{{T}}$ after correcting MC to reproduce data efficiency in $\eta$ and $\phi$. This resulted in a 0.2 % effect in the barrel and a 0.5 % effect in the endcap region.
- Dependence on pile-up interactions: as shown in Fig. 12, the efficiency has a small dependence on the number of pileup events in the end cap region, with about 1.0 % efficiency loss per 20 vertices. The MC simulation reproduces the effect well. This is accounted for by changing the distribution of the average number of pile-up interactions, resulting in a 0.1 % uncertainty.

The resulting uncertainties were added in quadrature to form the total systematic uncertainty.

**Fig. 13:**
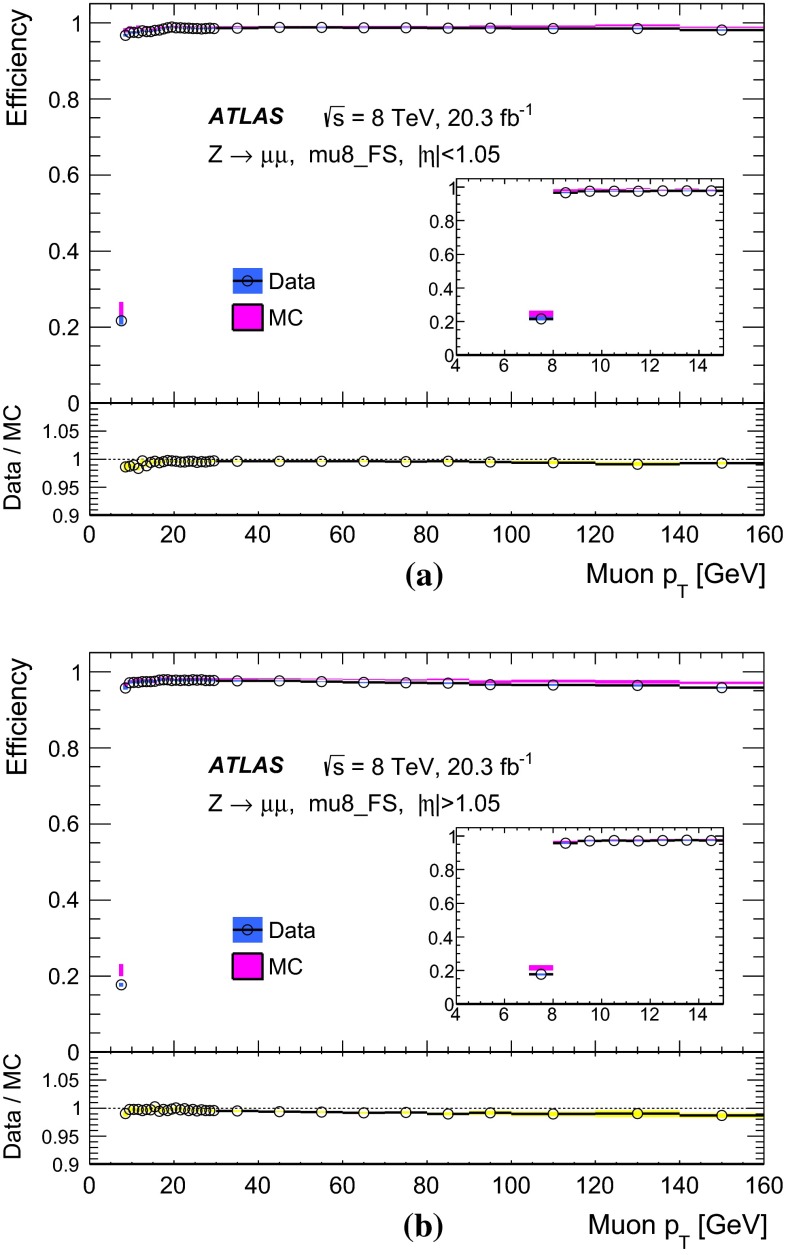
Efficiency of the event-filter full-scan mu8_FS as a function of the probe-muon transverse momentum $p_\mathrm{{T}}$, separately in **a** the barrel region and **b** the endcap region

Figure 13 shows the data and MC efficiencies for the mu8_FS trigger for the barrel and endcap regions. The efficiency plateaus for the barrel and endcap regions are 98.7 and 97.6 %, respectively. This results in a higher efficiency for the dimuon trigger than achieved by requiring two RoI-based single-muon triggers.

**Fig. 14:**
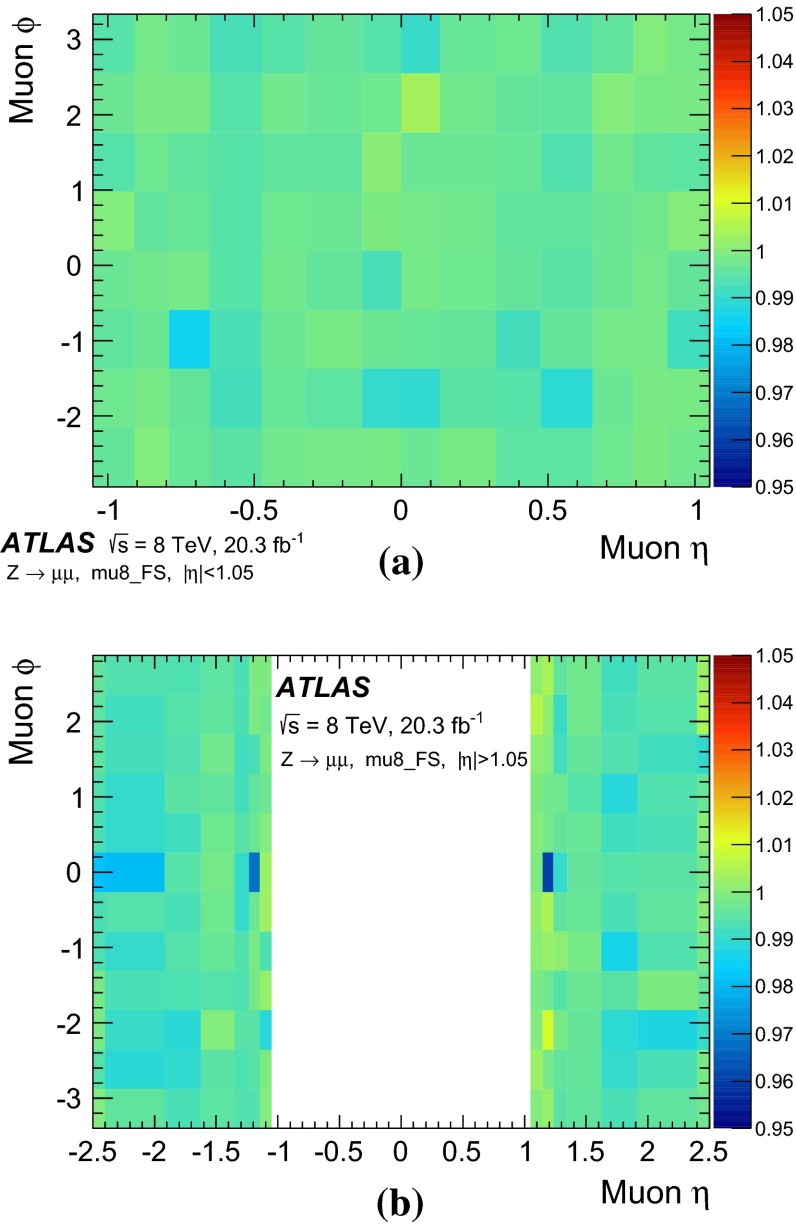
Ratio of the data and MC efficiencies for the mu8_FS trigger in bins of the probe-muon pseudorapidity $\eta$ and azimuthal angle $\phi$, in **a** the barrel region and **b** the endcap region

The ratio of the efficiencies in data and MC is shown as a function of $\eta$ and $\phi$ in Fig.  14 for the probe-muons with $p_{T}$ 10 GeV. It is consistent with unity to within 2 % except in two bins where the difference is as large as 5 %.

## Efficiency measurements at low $p_\mathrm{{T}}$

### Efficiency measurements with $J/\psi$

For the kinematic region of $p_\mathrm{{T}}\lesssim 10$ GeV, the efficiency was measured with the tag-and-probe method using $J/\psi$ meson decays.

A MC study shows that the efficiency is slightly dependent on the measured $d_0$. Therefore, the efficiencies of prompt and non-prompt muons can be different due to different $d_0$ distribution. This effect is mostly removed by the cuts on $d_0$ and $L_{xy}$ described in Sect. 3.5. The residual effect is then suppressed by reweighting the $d_0$ distribution to that of the prompt muons, which is obtained from the events with $L_{xy}< 0$.

Owing to a very high purity of the offline muon identification, the background also consists of muons, where the latter do not originate from the decay of a $J/\psi$ meson. The background fraction in the $J/\psi$ mass window is about 16 %, ranging between 13 and 20 % depending on the muon $p_\mathrm{{T}}$. The efficiency was measured by correcting the background effect using the side-bands of the invariant mass distribution.

### Systematic uncertainty

The following sources of systematic uncertainty were evaluated. The uncertainty numbers quoted in the following are for the efficiency measured as a function of the probe-muon $p_\mathrm{{T}}$ in the region of $4 < p_\mathrm{{T}}< 10$ GeV.

- Matching between probe-muon and trigger muon: the effect was estimated by relaxing the $\Delta R$ criterion from 0.12 to 0.15, and also by relaxing the $\Delta R$ distance cut between the two muons from 0.2 to 0.25. The estimated uncertainty is up to 3 % (2 %) at $p_\mathrm{{T}}= 4$ GeV in the barrel (endcap) region, decreasing to 1 % at $p_\mathrm{{T}}\gtrsim 6$ GeV.
- Reweighting of the $d_0$ distribution: the effect was estimated by comparing the efficiency with that obtained by not applying the $d_0$ reweighting. The estimated uncertainty is 1 % at $p_\mathrm{{T}}\sim 4$ GeV, decreasing to a negligible level at $p_\mathrm{{T}}\gtrsim 6$ GeV.
- Probe-muon charge dependence: the effect was estimated by comparing the efficiencies measured with positively charged and with negatively charged probe-muons. The estimated uncertainty is 1 % at low $p_\mathrm{{T}}\sim 4$ GeV, decreasing to 0.5 % at $p_\mathrm{{T}}\gtrsim 6$ GeV.
- Background contribution: the effect was estimated by not doing the background correction, resulting in a uncertainty of 0.1 %.
- Probe-muon selection criteria: the effect was estimated by changing typically by 10 % the thresholds of various selection criteria, leading to negligible effects.
- Dependence on pile-up interactions: Separate linear fits to the efficiency dependence on Nvtx in the data and MC simulation were performed in the range from Nvtx $=$ 5 to Nvtx $=$ 30 and extrapolated out to Nvtx $=$ 50. The dependence on the fit range was observed to be negligible. The largest difference between the fit results in data and MC simulation were observed to be 0.2 (0.4) % in the barrel (endcap). This difference is taken as the estimate of the resulting systematic uncertainty.

The total systematic uncertainties are obtained by adding the individual ones in quadrature.

### Low-$p_\mathrm{{T}}$ single-muon triggers

**Fig. 15:**
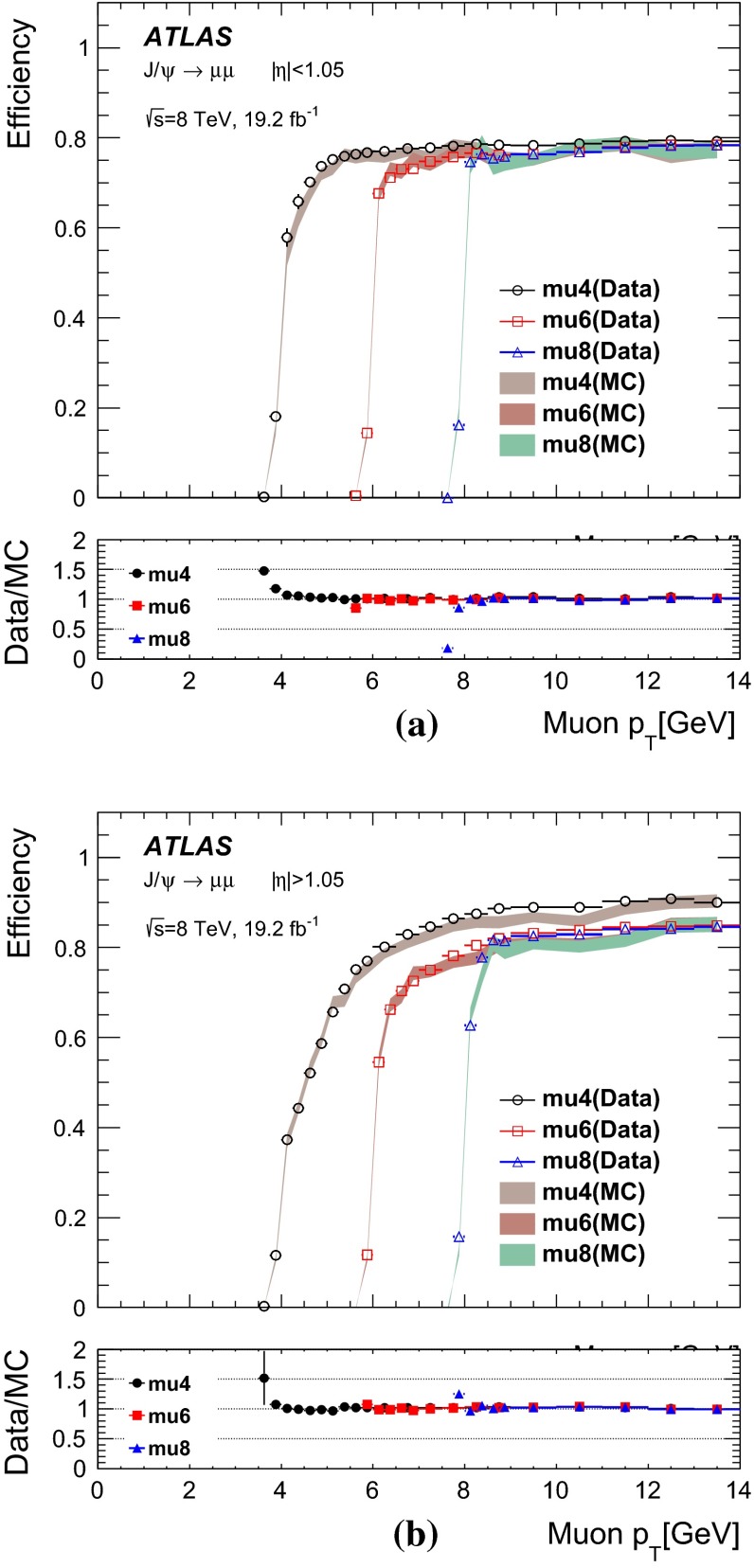
Efficiency of low transverse momentum $p_\mathrm{{T}}$ single-muon triggers, mu4, mu6 and mu8, as a function of the probe-muon transverse momentum $p_\mathrm{{T}}$ in **a** the barrel region and **b** the endcap region, in the data (*symbols*) and in the MC (*bands*). The *error bars* for MC indicate the statistical uncertainties only, while those for data indicate both the statistical and systematic uncertainties

Figure 15 shows the efficiency of the lowest-$p_\mathrm{{T}}$ single-muon triggers, mu4, mu6 and mu8 as a function of the $p_\mathrm{{T}}$ of the probe-muon.The efficiency of mu4 is about 40 % at the nominal threshold of 4 GeV. The mu4 turn-on curve rises slowly until $p_\mathrm{{T}}\sim 8$ GeV. The plateau efficiency of mu4 is higher by about 3 % in the endcap region, compared to those of mu6 and mu8.

**Fig. 16:**
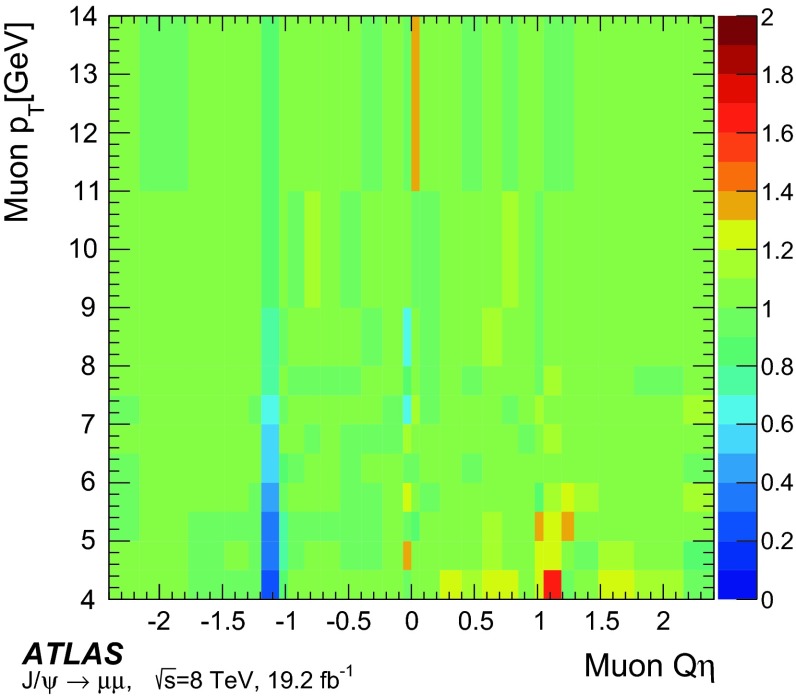
Ratio of the data and MC efficiencies of the mu4 trigger in bins of the probe-muon pseudorapidity $\eta$ multiplied by its charge, $Q\eta$, and the transverse momentum $p_\mathrm{{T}}$

The ratio of data and MC efficiencies of the mu4 trigger determined in bins of $p_\mathrm{{T}}$ and $Q\eta$, where $Q$ stands for the charge of the probe-muon is shown in Fig. 16.

The ratio is significantly lower than unity at $Q\eta \sim -1.1$ for $p_\mathrm{{T}}$ values up to $\sim 12$ GeV. In the muon spectrometer toroid magnetic field, the muons with $Q\eta >0$ ($<0$) bend toward the large (small) $|\eta |$ direction in the $r$–$z$ plane. The muons with $Q\eta \sim -1.1$ are thus likely to pass through only one layer of the RPC (see Fig. 1) and hence are not triggered. Figure 16 shows that this is not well modelled in the MC simulation.

## Efficiency measurements at very high $p_\mathrm{{T}}$

### Efficiency measurements with top quarks and $W$ associated with jets

For the kinematic region of $p_\mathrm{{T}}\gtrsim 100$ GeV, the efficiency was measured using muons from top quark and $W$ $+$ jet candidate events. Because they are statistically independent of each other and also correspond to background-enriched samples of each other, the efficiencies using muons in top quark and $W$ $+$ jet events can be obtained by solving the following two equations

$$\begin{aligned} \epsilon ^{t, \mathrm data}&= f^{t,\mathrm data}_{t} \epsilon _{t} + (1-f^{t,\mathrm data}_{t}) \epsilon _{W}, \\ \epsilon ^{W, data}&= f^{W, \mathrm data}_{W} \epsilon _{W} + (1-f^{W, \mathrm data}_{W}) \epsilon _{t}, \end{aligned}$$

where $\epsilon _{t(W)}$ is the efficiency in pure top quark ($W$ $+$ jets) events, and $\epsilon ^{t(W), \mathrm data}$ is the measured efficiency in the top quark ($W$ $+$ jets) sample. The factors $f^{t,data}_{t}$ and $f^{W,\mathrm data}_{W}$ denote the fraction of true top quark ($W$ $+$ jets) events in the top quark ($W$ with jets) sample, as determined by using MC simulation.

### Systematic uncertainty

In the following, sources of systematic uncertainty are discussed and the quoted uncertainty values are presented for the efficiency measured using the $W$ $+$ jets sample as a function of $p_\mathrm{{T}}$, in the region of $100 < p_\mathrm{{T}}< 400$ GeV.

- Muon isolation: to estimate this effect, the efficiency was measured by varying the isolation cut, both by loosening and by tightening the criteria, as well as by changing the $\Delta R$ cone size. The estimated uncertainty is typically 0.2 %;
- Muon–jet separation: the requirement on muon–jet separation serves also as an isolation cut. This effect was estimated by changing the $\Delta R$ criterion in the matching from 0.4 to 0.3 and 0.5. The estimated uncertainty is typically 0.1 and 0.3 % at maximum,
- $E_\mathrm{{T}}^\mathrm{{miss}}$ reconstruction: the effect was estimated by changing the threshold from 20 to 50 GeV, and also by introducing another tight cut of $E_\mathrm{{T}}^\mathrm{{miss}}\text {(calo)} > 120$ GeV. The estimated uncertainty is 0.5 % at maximum.
- Identification of $b$-jets: the effect was estimated by repeating the measurements with a different $b$-jet identification criterion, namely with 60 % efficiency and 80 % efficiency. The estimated uncertainty is typically less than 0.1 %.
- Cut on $p_\mathrm{{T}}^\mathrm{{jet}}$: the effect was estimated by raising the $p_\mathrm{{T}}^\mathrm{{jet}}$ threshold to 35 GeV. The estimated uncertainty is typically less than 0.1 %.
- Background contribution: the number of background events was estimated by using the dijet and $Z$ MC simulations and was found to be negligible at $p_\mathrm{{T}}> 100$ GeV.

All the contributions were added in quadrature to obtain the total systematic uncertainties.

### Single-muon trigger efficiency at $p_\mathrm{{T}}\gtrsim 100$ GeV

**Fig. 17:**
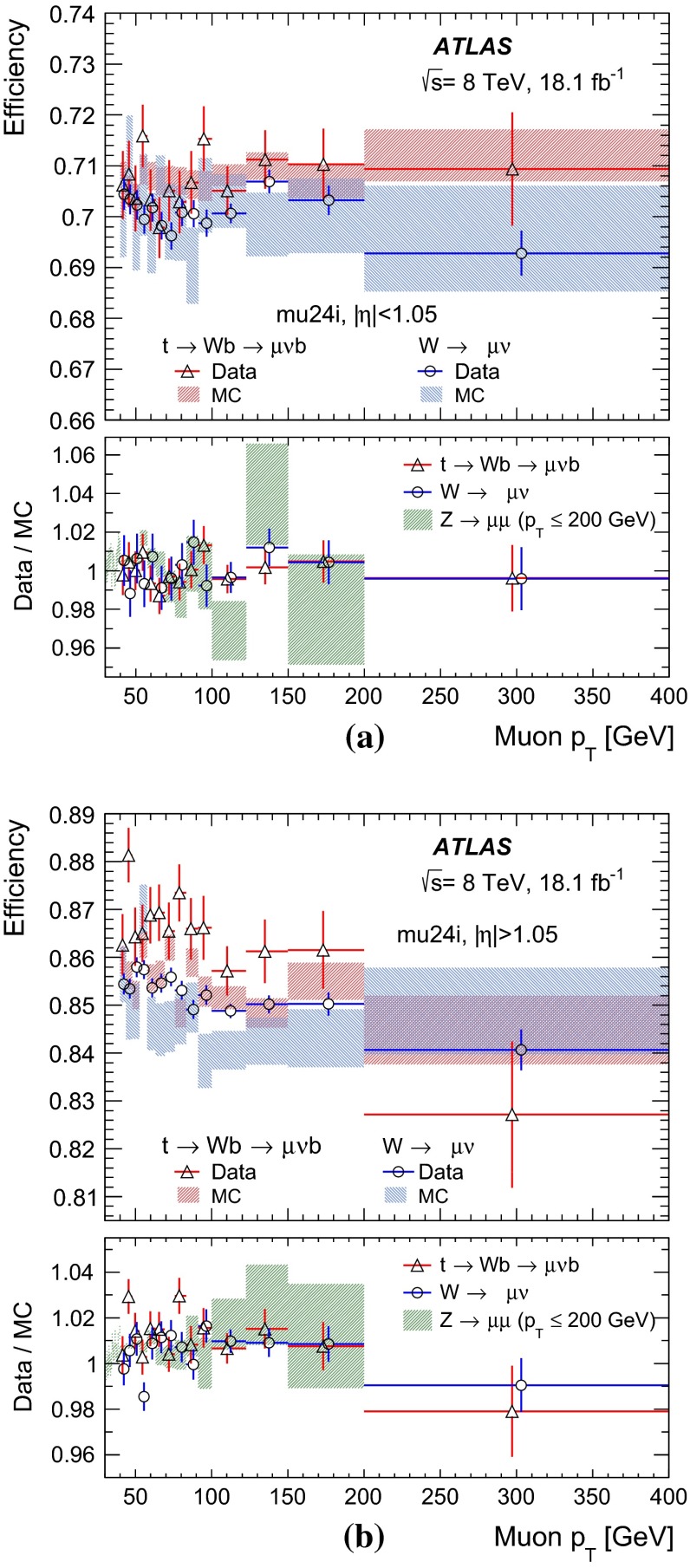
Efficiency of the mu24i trigger as a function of the probe-muon transverse momentum $p_\mathrm{{T}}$, as measured with the top quark and $W$ $+$ jet candidate events in the **a** barrel and **b** endcap regions. The *lower panels* show the ratio of the efficiencies in the data and MC simulation. Also shown is the efficiency as measured with the $Z$ decays using the tag-and-probe method. The *error bars* for MC simulation indicate the statistical uncertainties only while the *error bars* for data include both statistical and systematic uncertainties

Figure 17 shows the efficiencies measured using top quark and $W$ with jets events for the single isolated-muon trigger, mu24i, in the barrel and endcap regions as a function of the $p_\mathrm{{T}}$ of the probe-muon, up to $p_\mathrm{{T}}\sim 400$ GeV. The data and MC simulation agree well up to the very high $p_\mathrm{{T}}$ values.

Also shown in Fig. 17 are the ratios of the efficiencies in the data and MC simulation for the three samples used for the efficiency determination. The three measurements are in good agreement with each other throughout a large $p_\mathrm{{T}}$ range, providing a consistency check of the efficiency measurement in different physics processes with different experimental techniques and in the presence of different backgrounds.

The efficiency in the end cap is seen to drop off slightly at the highest $p_\mathrm{{T}}$ which is not observed in the barrel. This was further investigated and it was found that for the highest energy muons ($\approx 1000$ GeV) there is a slight loss of efficiency at the event-filter when combining the muon spectrometer and inner detector track. While the offline algorithm looks for large energy deposits in the calorimeter which arise from bremsstrahlung, the event-filter algorithm always uses a parameterised energy loss for a minimum ionising particle. Without correction, this can cause a mismatch in the momentum estimate in the inner detector and muon spectrometer causing the combination to fail. This occurs in the end cap where kinematically, for fixed $p_\mathrm{{T}}$, the energy of muons is much higher and thus high energy bremsstrahlung is more likely to occur. However, the effect is small, only occurs in the highest few $p_\mathrm{{T}}$ bins and accounts for a 4 $\%$ efficiency loss with a 2 $\%$ uncertainty.

## Conclusions

The ATLAS muon trigger has been successfully adapted to the challenging environment at the LHC such that stable and highly efficient data taking was achieved in the year 2012. The transverse momentum threshold for the single-muon trigger was kept at 24 GeV, with a well-controlled trigger rate of typically about 8.5 kHz at the Level-1 and 65 Hz at the event-filter. The processing times of the Level-2 and event-filter muon trigger algorithms were sufficiently short to fit within the computing resource limitations. The purity of the trigger is about 90 % at the event-filter, and more than half of the triggers originate from electroweak bosons production. The efficiencies are measured extensively with the proton–proton collision data at a centre-of-mass energy of 8 TeV. The systematic uncertainty in the measured efficiency for the single-muon trigger is evaluated to be about 0.6 % in a kinematic region of $25 < p_\mathrm{{T}}< 100$ GeV. The efficiency was measured over a wide $p_\mathrm{{T}}$ range (few GeV to several hundred GeV) by using muons from $J/\psi$ mesons, $Z$- and $W$-bosons, and top quark decays showing highly uniform and stable performance.

## Footnotes

1 Non-prompt muons are muons which originate from the decay of a secondary particle rather than coming directly from the primary $pp$ interaction.

2 Unless otherwise stated CP conjugate states are always implied.

3 ATLAS uses a right-handed coordinate system with its origin at the nominal interaction point (IP) in the centre of the detector and the $z$-axis along the beam pipe. The $x$-axis points from the IP to the centre of the LHC ring, and the $y$-axis points upward. Cylindrical coordinates $(r,\phi )$ are used in the transverse plane, $\phi$ being the azimuthal angle around the beam pipe. The pseudorapidity is defined in terms of the polar angle $\theta$ as $\eta =-\ln \tan (\theta /2)$.

4 The term prescale means that only one in N events passing the trigger is accepted at that trigger level, where N is an integer definite number called the prescale factor. At Level-1 every Nth event is accepted. At the high level trigger a random number generator is utilised such that one out of every N events is accepted.

5 Transverse mass is defined as $m_{T}^{2} = m^{2} + p_{x}^{2} + p_{y}^{2}$ and has the useful propriety that it is invariant under Lorentz boosts along the beam direction.

6 This trigger was active but with a prescale factor of 10.

7 The functional form is $\frac{a}{1+\exp {\{b(c-p_\mathrm{{T}})\}}}$, where $a$ indicates the plateau value, $b$ the steepness of the turn-on slope, and $c$ the threshold value.
